# Time of Emergence and Large Ensemble Intercomparison for Ocean Biogeochemical Trends

**DOI:** 10.1029/2019GB006453

**Published:** 2020-08-23

**Authors:** Sarah Schlunegger, Keith B. Rodgers, Jorge L. Sarmiento, Tatiana Ilyina, John P. Dunne, Yohei Takano, James R. Christian, Matthew C. Long, Thomas L. Frölicher, Richard Slater, Flavio Lehner

**Affiliations:** ^1^ Program in Atmospheric and Oceanic Sciences Princeton University Princeton NJ USA; ^2^ Center for Climate Physics Institute for Basic Science Busan South Korea; ^3^ Pusan National University Busan South Korea; ^4^ Max Plank Institute for Meteorology Hamburg Germany; ^5^ NOAA Geophysical Fluid Dynamics Laboratory Princeton NJ USA; ^6^ Los Alamos National Laboratory Los Alamos NM USA; ^7^ Canadian Center for Climate Modeling and Analysis Victoria British Columbia Canada; ^8^ National Center for Atmospheric Research Boulder CO USA; ^9^ Climate and Environmental Physics, Physics Institute University of Bern Bern Switzerland; ^10^ Oeschger Centre for Climate Change Research University of Bern Bern Switzerland

**Keywords:** ocean biogeochemistry, Time of Emergence, Earth system models, model intercomparison, uncertainty quantification, anthropogenic trends

## Abstract

Anthropogenically forced changes in ocean biogeochemistry are underway and critical for the ocean carbon sink and marine habitat. Detecting such changes in ocean biogeochemistry will require quantification of the magnitude of the change (anthropogenic signal) and the natural variability inherent to the climate system (noise). Here we use Large Ensemble (LE) experiments from four Earth system models (ESMs) with multiple emissions scenarios to estimate Time of Emergence (ToE) and partition projection uncertainty for anthropogenic signals in five biogeochemically important upper‐ocean variables. We find ToEs are robust across ESMs for sea surface temperature and the invasion of anthropogenic carbon; emergence time scales are 20–30 yr. For the biological carbon pump, and sea surface chlorophyll and salinity, emergence time scales are longer (50+ yr), less robust across the ESMs, and more sensitive to the forcing scenario considered. We find internal variability uncertainty, and model differences in the internal variability uncertainty, can be consequential sources of uncertainty for projecting regional changes in ocean biogeochemistry over the coming decades. In combining structural, scenario, and internal variability uncertainty, this study represents the most comprehensive characterization of biogeochemical emergence time scales and uncertainty to date. Our findings delineate critical spatial and duration requirements for marine observing systems to robustly detect anthropogenic change.

## Key Points

- Anthropogenic changes in sea surface temperature and air‐sea CO_2_ fluxes emerge decades prior to changes in the biological carbon pump, ocean color, and sea surface salinity
- Detecting anthropogenic changes in ocean biogeochemistry requires sustained monitoring from observing systems with large spatial footprints
- Internal variability, model uncertainty, and scenario uncertainty are all important sources of uncertainty for projecting future changes in ocean biogeochemistry

## 1 Introduction

The ocean's physical and biogeochemical state determine its habitability and capacity for sequestration of anthropogenic carbon. Rising temperatures, changing circulation, and acidification impact marine ecosystems and alter the cycling of carbon within the ocean (Bopp et al., 2013; Frölicher et al., 2016; Lovenduski et al., 2016; Riebesell et al., 2009; Sarmiento et al., 1998); however, the timing and magnitude of these potential impacts is uncertain due to uncertainty in the projections of Earth System Models (ESMs), global climate models that include an interactive representation of the global carbon cycle. Uncertainties in these projections stem from the following three sources: (1) the structural uncertainty associated with the different models used to make projections, (2) the scenario uncertainty associated with different future emission pathways, and (3) the natural internal variability uncertainty associated with natural fluctuations of the coupled climate system (Deser et al., 2012; Hawkins & Sutton, 2009). Here, we assess the contributions of these three types of uncertainty to the timing and magnitude of changes in key ocean parameters. We use multiple ESMs, each with multiple realizations and multiple emissions scenarios, to partition uncertainty, thereby providing the first multimodel assessment of ocean biogeochemistry to address the three sources of uncertainty within a consistent framework.

We focus the first part of our analysis on the Time of Emergence (ToE) diagnostic, which represents the time scale over which an anthropogenic or forced trend in the climate system emerges above the uncertainty induced by natural internal variability. ToE is a multipurpose metric which stands to (1) inform observing system design by providing a baseline for monitoring time and space scales required for trend detection, (2) inform impacts research because impacts on organisms and ecosystems are likely to manifest most strongly once anthropogenically induced trends exceeds the envelope of natural variability to which biota are adapted, and (3) normalize anthropogenic responses relative to their natural internal variability, allowing for comparison across disparate variables, across different ESMs, and across a spectrum of forcing scenarios, providing a framework for model, scenario, and impact intercomparison.

ToE can be estimated with projections made from initial condition Large Ensemble (LE) experiments of ESMs (Long et al., 2016; McKinley et al., 2016; Rodgers et al., 2015; Schlunegger et al., 2019). The central idea with LEs is that the initial conditions of a climate simulation only need tiny perturbations for climate variability to quickly randomize between the ensemble members for any particular time slice. Differences between projections of ensemble members are solely due to natural internal variability, so natural internal variability can be identified as the difference between ensemble members at any point in time and the forced response to anthropogenic modification of the climate system (e.g., greenhouse gas [GHG] emissions and land use change) can be identified through the common response, or average of the ensemble members (e.g., Deser et al., 2014). Over the duration of the projection, the magnitude of the forced response (signal) may become statistically distinguishable from natural internal variability (noise). This point in time defines the ToE.

Previous studies have shown that anthropogenic changes in different ocean properties exhibit vastly different time scales of emergence (Christian, 2014; Frölicher et al., 2016; Henson et al., 2016, 2017; Keller et al., 2014; Rodgers et al., 2015; Schlunegger et al., 2019). Schlunegger et al. (2019) identified the mechanistic controls leading to disparate emergence time scales for the forced signals in the ocean carbon cycle. Using an LE of a single ESM (GFDL‐ESM 2M), they find variables that represent the integrated effect of invading anthropogenic carbon into the global ocean, such as pH, emerged most rapidly, with ToEs of only a few years. Within a few decades, changes in sea surface temperature (SST) and in air‐sea CO_2_ fluxes emerge. Changes in the physical state of the upper ocean, including upper ocean mixing, and associated changes in biological processes, such as the export of organic matter which depends on nutrients supplied through mixing, only emerge after many (5+) decades.

Here, for the first time, we use multiple LEs of different ESMs with different forcing scenarios to determine if this chronology of emergence is a robust feature of current‐generation ESMs and to determine the impacts of mitigation on emergence time scales, which depend on both the forced signal and the forcing‐dependent internal variability. As a complement to the presentation of ToE, we also include confidence intervals for emergence (signal‐to‐noise ratios [SNRs]) over the observational period (~1990 to present).

As a second component of the analysis, we utilize the multiple LEs and multiple scenarios to partition contributions from sources of uncertainty—scenario, model, internal—in projections of change in the ocean state. Previous studies that evaluated uncertainty in projections of ocean biogeochemical variables have a number of inconsistencies in terms of the definition of noise (e.g., preindustrial vs. contemporary, single‐model vs. CMIP5 mean) and filtering to retrieve the forced signal (e.g., temporal smoothing techniques vs. fourth‐order polynomial fitting), complicating comparisons across studies. A central objective here is to take advantage of the opportunity offered by the multi‐LE approach to provide a consistent and unifying framework for trend detection and evaluating projection uncertainty.

We focus the analysis on five observable or observationally constrained biogeochemical variables that impact the cycling of carbon within the global ocean: SST, air‐sea CO_2_ flux, export of organic carbon from the surface ocean to depth, sea surface chlorophyll, and sea surface salinity (SSS). SST, observable by satellite, ships, and buoys, impacts carbon through setting the solubility of CO_2_, and through the temperature dependence of biological activity and the biologically mediated export of carbon to depth; it is also indicative of state‐dependent density stratification, vertical exchange, and overturning circulation. The flux of CO_2_ at the air‐sea interface, which allows for the invasion of anthropogenic carbon into the global ocean, is estimated from shipboard surface pCO_2_ measurements and interpolation in space and time using known or empirical relationships with more densely observed oceanographic properties like SST, sea surface height (SSH), and ocean color (Rödenbeck et al., 2015) or through ocean or atmospheric inversion methods (Wanninkhof et al., 2013).

The biological export of organic carbon to depth (export production) and the ocean observable from which it is derived or estimated, ocean color, an indicator of algal biomass, are relevant to the ecologically important transfer of energy from the base of the marine food web and the associated climatologically important transfer of carbon from the upper ocean to depth (Siegel et al., 2016). Finally, SSS, monitored via remote sensing (since year 2010; Font et al., 2013), the Argo program (since Year 2000; Riser et al., 2016), and shipboard measurements (reliably since the 1970s; Bingham et al., 2002), provides a means to monitor the climate change impact on freshwater fluxes and ocean circulation, important drivers of carbon cycling in the ocean.

Numerous observational programs with the intended goal of monitoring changes in the ocean's carbon cycle are currently underway, and observational records extend 20–30 yr. We note in particular (1) the Regional Carbon Cycle Assessment Project (RECCAP; Wanninkhof et al., 2013) for which the last phase of analysis focused on air‐sea CO_2_ fluxes over the 20 yr period 1990–2009 and forthcoming analysis will focus on the 30 yr period 1990–2019, and (2) ocean color observations, for which 20 yr of continuous coverage and resulting synthesis products are presently available (e.g., Lavender et al., 2015). In the second section of this work, as a means to directly facilitate interpretation of the observational record, we utilize the multiple LEs to provide confidence intervals for emergence of anthropogenic signals over these observational periods.

## 2 Methods

### 2.1 Models

LE simulations have been conducted separately with four Earth system models: (i) CanESM2, (ii) CESM1‐BGC, (iii) GFDL‐ESM2M, and (iv) MPI‐ESM‐LR. CanESM2 is described in Arora et al. (2011), Christian (2014), Christian et al. (2010), and Zahariev and Christian (2008). The LE suite with CESM1 is described by Kay et al. (2015) with the marine biogeochemistry model described by Long et al. (2013), Moore et al. (2013), and Lindsay et al. (2014). ESM2M is described by Dunne et al. (2012, 2013) with the LE documented in Rodgers et al. (2015). The MPI‐ESM‐LR is described by Giorgetta et al. (2013) with the marine biogeochemistry model described by Ilyina et al. (2013) and the LE first described in Bittner et al. (2016) and Li and Ilyina (2018) and formally described in Maher et al. (2019).

The models have a number of important similarities, including non‐eddy‐permitting ocean resolution, surface ocean carbonate chemistry broadly following standard protocols (OCMIP2; Najjar et al., 2007), and general ecological complexity. However, there are also many important differences, types of phytoplankton represented, whether or not chlorophyll is explicitly and interactively computed, and the particle aggregation, remineralization and sinking schemes.

### 2.2 LE Experimental Design and Initialization

For each of these models, at least 30 ensemble members have been run over the historical period with historical forcing spanning 1950–2005, and extended though at least 2099 with RCP8.5 (high‐emissions) forcing. For each of these models, an RCP4.5 (moderate‐emissions) extension was also performed, with either a large (>30 members, GFDL and MPI), medium (9–15 members, CESM1) or small (5 members, CanESM2) ensemble. Output from each LE was regridded to a common horizontal 1°x1° ocean grid.

Each of the four LEs was initialized in a unique way, but with a common goal of producing at least 30 realizations of the climate, each realization experiencing a different, equally probable sequence of internal climate variability. The CanESM2 LE was initialized in two phases. First, five members, which covered the period 1850–2005, were generated from initial conditions chosen from different years of the preindustrial control runs. Second, each of the five members were branched into 10 members at Year 1950 through slight modification of the atmospheric initial conditions, achieved through changing the seed of a random‐number generator within the cloud parameterization (Kirchmeier‐Young et al., 2017). In this study, all calculations are done using only 30 members for RCP8.5. The RCP4.5 scenario consist of a small, five‐member ensemble which spans the time period 2006–2100.

The CESM LE was also initialized through microperturbation of the atmospheric initial conditions at Year 1920 of ensemble member Number 1, achieved through random, round‐off error (10^−14^) differences to the initial air temperature field for 30+ members (Kay et al., 2015). A few members of the Large and Medium ensembles did not simulate ocean biogeochemistry. For the RCP8.5 CESM LE, we use the first 30 members for which ocean biogeochemistry was available, and for the RCP4.5 ME there are only 9 members with biogeochemical variables available and 15 members with physical variables. The RCP4.5 ME spans the time period 2005–2080 (Sanderson et al., 2018).

The GFDL LE was initialized through modest perturbation to the initial climate state (ocean, atmosphere, land, and sea ice). The first ensemble member was branched into 29 additional members at Year 1950, using 2–30 January 1950 of the first ensemble member for the initial conditions of Members 2–29 (Rodgers et al., 2015). The 30 members cover the historical period (1950–2005) and at Year 2006 are branched into RCP8.5 and RCP4.5 extensions which span the time period 2006–2100.

The MPI LE was initialized through macroperturbations to the initial climate state. Different years of the preindustrial control run were used to initialize the climate state of 100 realizations which span the time period 1850–2005, with 100‐member RCP8.5 and RCP4.5 extensions which span the time period 2006–2099 (Maher et al., 2019). In this study, all calculations are done using only the first 30 members.

### 2.3 ToE Calculations

ToE denotes the time at which a signal of interest is statistically distinguishable from background noise. The signal of interest in this work is the anthropogenic or “forced trend,” and the noise is natural or internal variability. The forced signal represents the common trend among the ensemble members, and the noise represents the variation among ensemble members. By design, variation among the ensemble members can only be due to natural internal variability. For each LE, the signal is the ensemble average trend, computed as the average of the ~30 ensemble members' trends (linear, least squares trend). The noise is the ensemble standard deviation, computed as the standard deviation of the ~30 ensemble member trends.

The “signals” produced from natural internal variability are approximately normally distributed around the ensemble mean, and will average to zero deviations from the ensemble mean over a long enough time horizon or with sufficient ensemble members. Therefore, a standard two‐sided Student's *t* test is used to test whether a given signal could be explained by natural variability alone. The null hypothesis (that the signal could be due to natural variability) is rejected with >95% confidence when the magnitude of the signal (forced trend) is twice the magnitude of the noise (natural internal variability), that is, when the SNR equals or exceeds 2. The ToE is the first year at which SNR ≥ 2. All trend calculations are performed on annual means and started in Year 1990, as this is the approximate beginning of the observing era for ocean biogeochemistry (Woods, 1985).

ToE calculations are performed at the grid cell level (1° × 1°), regionally, and globally. For global and regional ToE calculations, first a single time series of the domain‐averaged or integrated quantity is taken, providing ~30 (or fewer for RCP4.5 simulations) individual time series. From these individual time series (either local, regional, or global) the trends, signal, noise, and ToE are computed.

The regional bounds from the RECCAP protocol (http://www.globalcarbonproject.org/reccap/protocol.htm ) are used for regional analysis (Figure S2). The Southern Ocean is defined as waters south of 44°S. The Arctic is defined as the region north of 65°N. For the Pacific and Atlantic basins, north is defined as 18–65°N, equatorial is defined as 18°N to 18°S, and south is defined as 18°S to 44°S. For the Indian basin, north is defined as lying north of 0°N, and south is defined as 44–0°S.

### 2.4 Partitioning Uncertainty

In this section we expand upon the characterization of the three sources of uncertainty inherent to climate projections (model, scenario, and internal variability) and formalize how we estimate their magnitudes.

First, model or structural uncertainty in projections results from imperfect model representation of the Earth system. The approximately 20 state‐of‐the‐art ESMs from different modeling centers internationally have different model constructions (i.e., different physical and biogeochemical representation and parameterizations) and as a consequence project different deterministic responses to anthropogenic forcing. For example, under a high‐emissions scenario (RCP8.5), end of century global mean annual temperature change relative to preindustrial ranges from 3.2°C to 5.4°C for the full suite of climate models (Collins et al., 2013). These differences among ESM responses to anthropogenic forcing give a lower bound on the model or structural uncertainty inherent to projection. This is considered a lower bound because the actual uncertainty could be greater, as models are not independent and may share biases, artificially reducing their disagreement.

By virtue of working with LEs, we are able to improve on or refine the methods considered in the original definition of model uncertainty (e.g., Hawkins & Sutton, 2009). First, we take the ensemble mean of each ESM's LE, for both the RCP8.5 and RCP4.5 scenario, isolating the forced response of each ESM.

(1) $${\mathrm{LE}}_{m,r85,\text{forced}}\left(t\right)=\frac{\sum_{1}^{n}{\mathrm{LE}}_{m,r85}\left(t\right)\;}{n}\;$$

(2) $$\begin{array}{l} {\mathrm{LE}}_{m,r45,\text{forced}}\left(t\right)=\frac{\sum_{1}^{n}{\mathrm{LE}}_{m,r45}\left(t\right)}{n}\; \end{array}$$

where *m* indicates the ESM, *r85* and *r45* indicate RCP8.5 and RCP4.5 scenarios, respectively, *n* is the number of ensemble members for given model and scenario, and *t* is the years between 2000 and 2100. The transition from historical to scenario forcing occurs at Year 2006; therefore, the historical experiments are used to populate Years 2000–2005 for both *LE*_*m,r85*_ and *LE*_*m,r45*_.

From here, we define model uncertainty (*U*_*M*_) as simply the range generated by the four ESM's forced time series (*LE*_*m,r85,forced*_) or the difference between the minimum and maximum *LE*_*m,r85,forced*_ for the RCP8.5 forcing scenario.

(3) $$U_{M}\left(t\right)=\mathrm{Max}\left[{\mathrm{LE}}_{m=1:4,\text{forced},r85}\left(t\right)\right]‐\mathrm{Min}\left[{\mathrm{LE}}_{m=1:4,\text{forced},r85}\left(t\right)\right]\;$$

where *t* is the years between 2000 and 2100 and *m* denotes the four ESMs, of which the maximum and minimum are taken for each time step, and their difference used to define *U*_*M*_. The ensemble mean from a LE (*LE*_*m,r85,forced*_) gives the forced signal of the given model—eliminating the need to fit a polynomial or assume a distribution, as was necessary methodology in pre‐LE studies (Hawkins & Sutton, 2009). This has proven to be particularly important at local to regional scales when considering quantities with high variability (Deser et al., 2020; Lehner et al., 2020). We use the RCP8.5 LEs for two reasons: (1) The larger forcing that persists through the century will reveal model differences more effectively than the moderate‐ or aggressive‐mitigation forcing scenario, and (2) this is the scenario with the most ensemble members available, at least 30 members for each ESM. We note that using only four ESMs is an underrepresentation of the full model uncertainty; however, we discuss in the results section that for SST, air‐sea CO_2_ flux and primary production, the suite of four models is representative of the spread of the forced response demonstrated by the larger suite of CMIP5 models considered in Bopp et al. (2013) and Jones et al. (2013).

Second, scenario uncertainty arises due to uncertainty in the pathway of future emissions of GHGs and other climactically important constituents. To represent this uncertainty, standardized representative concentration pathways (RCPs), which prescribe the evolution of atmospheric GHGs, aerosols, and land use change, among other factors, have been developed for use by the climate modeling community (Moss et al., 2010). The RCPs are constructed to provide a specific and consistent radiative imbalance throughout the century, with no‐mitigation scenario, RCP8.5, producing 8.5 W/m^2^ imbalance at Year 2100. Two moderate‐emissions scenarios, RCP6.0 and RC4.5, and a low‐emission scenario, RCP2.6, complete the suite of four RCPs. Examining differences between projections using various emissions scenarios provides an estimate of scenario uncertainty. Scenario uncertainty (U_S_) is often defined as the difference between the highest (RCP8.5) and lowest (RCP2.6) emission scenarios. However, we use the differences between the RCP8.5 and RCP4.5 multi‐LE mean (the mean of the four LE means) for each scenario, as numerous ensemble members for the RCP2.6 scenario are not available for all the ESMs considered in this work.

We estimate scenario uncertainty by first computing the RCP8.5 multiensemble mean and the RCP4.5 multiensemble mean,

(4) $${\bar{\mathrm{LE}}}_{\text{forced},r85}\left(t\right)=\frac{\sum_{1}^{m}{\mathrm{LE}}_{m,\text{forced},r85}\left(t\right)}{m}\;$$

(5) $${\bar{\mathrm{LE}}}_{\text{forced},r45}\left(t\right)=\frac{\sum_{1}^{m}{\mathrm{LE}}_{m,\text{forced},r45}\left(t\right)\;}{m}\;$$

where $\bar{\mathrm{LE}}$_*forced,r85*_ and $\bar{\mathrm{LE}}$_*forced,r45*_ are the multiensemble mean for the RCP8.5 and RCP4.5 scenarios receptivity, *t* are the years between 2006 and 2100, and *m* denotes the four ESMs. Scenario uncertainty (*U*_*S*_) is then computed as the difference between the RCP8.5 and RCP4.5 multiensemble means.

(6) $$U_{S}\left(t\right)={\bar{\mathrm{LE}}}_{\text{forced},r85}\left(t\right)‐{\bar{\mathrm{LE}}}_{\text{forced},r45}\left(t\right)$$

where *t* is the years between 2006 and 2100.

Finally, we consider natural internal variability uncertainty, the uncertainty stemming from intrinsic, internal climate variability. We compute natural internal variability uncertainty (*U*_*IV*_) for each of the ESMs. We define *U*_*IV*_ as spread between the minimum and maximum ensemble member at a given year.

(7) $$U_{\mathrm{IV},m}\left(t\right)=\mathrm{Max}\left[{\mathrm{LE}}_{m,r85,e=1:30}\left(t\right)\right]‐\mathrm{Min}\left[{\mathrm{LE}}_{m,r85,e=1:30}\left(t\right)\right]$$

where *t* is the years between 2000 and 2100, *m* denotes the four ESMs, and *e* denotes the ensemble members in each ESM's LE. By virtue of using a LE, rather than a single ensemble member or preindustrial control run from one or many models, our definition of natural internal variability uncertainty differs from previous studies Hawkins and Sutton (2009). Our definition allows for changes in natural internal variability over time and does not require that we assume a distribution form to define internal variability. We use the RCP8.5 LEs to define internal variability as this is the scenario with the most ensemble members available for each of the LEs.

To partition sources of uncertainty, we expand upon the methodology developed in Frölicher et al. (2016), for which total uncertainty (*U*_*T*_) is the linear sum of model, scenario, and internal variability uncertainty.

(8) $$U_{T}\left(t\right)=U_{M}\left(t\right)+U_{S}\left(t\right)+\mathrm{Max}\left[U_{\mathrm{IV},m=1:4}\left(t\right)\right]$$

where *t* is the years between 2000 and 2100, and the largest internal variability of the four ESMs is chosen to represent internal variability. This total uncertainty is *not* computed to evaluate absolute uncertainty or uncertainty contributions but rather for the purpose of estimating fractional contributions to projection uncertainty from model, scenario and internal variability uncertainty.

Partitioning uncertainty into the different sources is done by dividing the individual sources (model, scenario, and internal) by the total uncertainty to yield the model uncertainty contribution (*UC*_*M*_), scenario uncertainty contribution (*UC*_*S*_), and internal variability uncertainty contribution (*UC*_*IV*_).

(9) $${\mathrm{UC}}_{M}\left(t\right)=\frac{U_{M}\left(t\right)}{U_{T}\left(t\right)}\;$$

(10) $${\mathrm{UC}}_{S}\left(t\right)=\frac{U_{S}\left(t\right)\;}{U_{T}\left(t\right)}$$

(11) $${\mathrm{UC}}_{\mathrm{IV}}\;\left(t\right)=\frac{\mathrm{Max}\left[U_{\mathrm{IV},m=1:4}\left(t\right)\right]\;}{U_{T}\left(t\right)}$$

We also define the internal variability uncertainty contribution for each of the four ESMs (*UC*_*IV,m*_), and the range of the four individual *UC*_*IV*_ estimates is used to estimate the structural uncertainty inherent to defining internal variability uncertainty (Δ*UC*_*IV*_).

(12) $${\mathrm{UC}}_{\mathrm{IV},m}\left(t\right)=\frac{U_{\mathrm{IV},m}\left(t\right)}{U_{T}\left(t\right)}\;$$

(13) $$\Delta {\mathrm{UC}}_{\mathrm{IV}}\;\left(t\right)=\frac{\mathrm{Max}\left[U_{\mathrm{IV},m=1:4}\left(t\right)\right]‐\mathrm{Min}\left[U_{\mathrm{IV},m=1:4}\left(t\right)\right]}{\;U_{T}\left(t\right)}$$

The magnitude of Δ*UC*_*IV*_ characterizes known uncertainty in the extent to which internal variability challenges climate projections.

## 3 Results

### 3.1 Signals and ToE

#### 3.1.1 Mean State Changes

**Figure 1:**
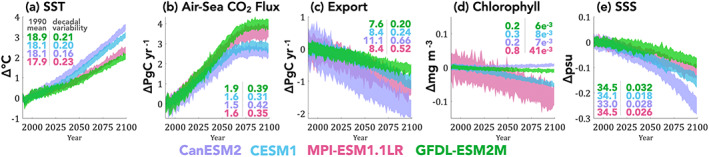
Global annual changes under historical RCP8.5 forcing for each ESM relative to Year 1990 for (a) global mean sea surface temperature (SST), (b) globally integrated air‐sea CO_2_ flux, (c) export production, (d) surface chlorophyll concentration, and (e) sea surface salinity (SSS). Values given in the left‐hand column are global mean in Year 1990 and in the right‐hand column are the 95% confidence intervals for magnitude of internal variability in globally integrated decadal trends for each LE in units of (a) °C/decade, (b, c) Pg C/decade, (d) mg Chl/m^3^/decade, and (e) practical salinity units/decade. Larger values indicate stronger decadal variability over the global domain.

The four ESMs considered span the CMIP5, RCP8.5 transient climate response given in Bopp et al. (2013) of between 2°C (GFDL) to 3.5°C (CanESM2) warming by end of century, relative to Year 1990 temperatures (Figure 1a). The four ESMs considered also span the RCP8.5 CMIP5 range of 21st century air‐sea CO_2_ flux given in Lovenduski et al. (2016) for which the ocean takes up an additional 2.5 Pg C/yr (CanESM2) to 4 Pg C/yr (GFDL) by the end of the 21st century (Figure 1b).

Global export production is projected to decline for all ESMs; however, the magnitude of decline is model‐dependent (Figure 1c). For export production, declines range between 0.5 and nearly 2.0 Pg C/yr by the end of the 21st century. Detailed attribution for the decline in export production for CESM1 and ESM2M can be found in Laufkötter et al. (2016).

Globally averaged surface chlorophyll concentrations are projected to decline for all models (Figure 1d) except for CanESM2, for which global chlorophyll concentrations increase modestly, as a residual of regionally heterogeneous trends (supporting information Figures S2 and S7g). Pronounced variability and decline of surface chlorophyll in the MPI‐ESM‐LR is related to a mean state bias (4 times higher chlorophyll concentrations relative to the other three models). For CanESM2, the rise in globally averaged surface chlorophyll concentrations occurs despite the decrease in biological export, adding complexity to the application of the ocean color record to estimate export production.

The globally averaged surface ocean is projected to freshen in all four ESMs over the 21st century (Figure 1e), consistent with CMIP5 projections. Net freshening occurs due to increased precipitation (freshening) over the Pacific basin overwhelming increased evaporation (salinification) of Atlantic basin (Levang & Schmitt, 2015). The magnitude of global freshening scales with the magnitude of sea surface warming for the four ESMs considered here.

#### 3.1.2 Global and Regional ToE

**Figure 2:**
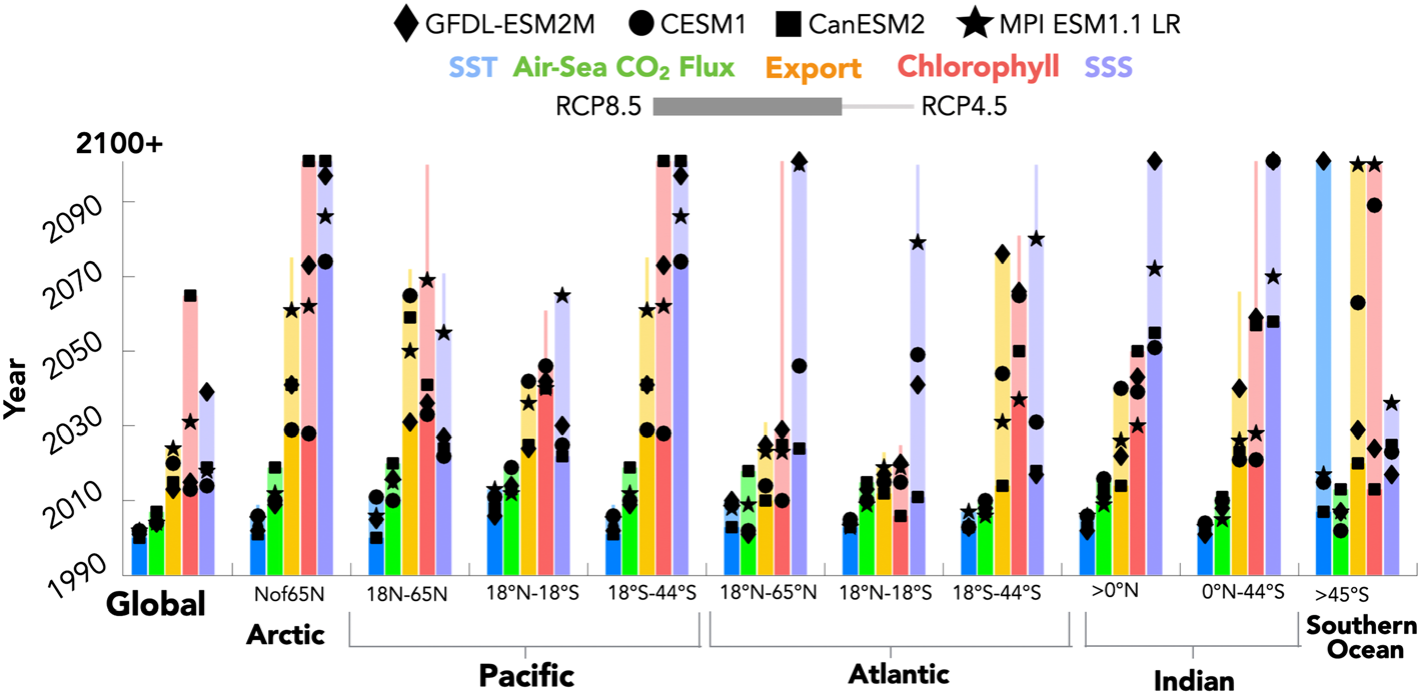
Global and regional Time of Emergence for SST, air‐sea CO_2_ flux, export production, surface chlorophyll, and SSS. For each variable and domain, the length of the bar indicates year after Year 1990 for which the anthropogenic signal is first emergent above natural internal variability for the model with the latest emergence time. For RCP8.5, symbols for each model are located at the year of emergence for the given variable and region. Thick bars indicate emergence times for RCP8.5, and thin bars indicate the range of emergence times for RCP4.5. For RCP8.5, each bar's color hue is lightened between the minimum and maximum ToE to highlight the spread between the models ToE estimates. The thin extensions from some of the variables and locations indicate emergence times that are longer for RCP4.5 forcing than for RCP8.5 forcing. Otherwise, the emergence times are equivalent for the two scenarios, and therefore the RCP4.5 forcing ToE is not visible. Model symbols are not shown for the RCP4.5 scenario.

We now consider when these global and regional anthropogenic changes emerge from natural internal variability for the four ESMs. The ToEs, referenced to the Year 1990, are given for SST, air‐sea CO_2_ flux, export production, surface chlorophyll concentrations, and SSS (Figure 2). Globally, and for most regions, anthropogenic trends in SST emerge between 10 and 20 yr, followed by anthropogenic trends in air‐sea CO_2_ flux (between 20 and 30 yr). The Southern Ocean is the only region with significant model disagreement in the timing of SST emergence. This is due to GFDL's ESM2M model, which projects a weak cooling trend over this region (Figure S5e). ESM2M does not emerge during this century and thus disagrees with the ~20–30 yr emergence times for significant warming estimated by the other LEs (Figure S5).

Air‐sea CO_2_ exchange is the variable with highest degree of ToE agreement between the LEs. All ESMs are within approximately a decade of each other, even for hot spots of variability such as the Southern Ocean, the North Atlantic, and the equatorial Pacific. The ESMs agree that emergence of global signals should occur within ~15 yr, and for most regional signals within 15–30 yr, relative to Year 1990. For air‐sea CO_2_ fluxes and SST, the global and regional emergence times for the RCP4.5 and RCP8.5 scenarios are equivalent as emergence for these variables occurs before the forcing scenarios diverge (Meinshausen et al., 2011).

Global changes in export production emerge between 25 and 40 yr, relative to Year 1990. However, regional changes have longer time scales of emergence (40–110+ yr) and larger disagreement between ESMs (20 to 60+ yr disagreement depending on region). Surface chlorophyll concentrations also take many decades to emerge, consistent with Henson et al. (2010), and the model spread is even greater than for export. For most regions, surface chlorophyll emergence times lag export production emergence time scales by a few years to a decade.

Global freshening emerges within 20–30 yr for CESM1, CanESM2, and MPI‐ESM‐LR and within 50 yr for GFDL, relative to Year 1990. Regional emergence times, even for areas of stronger salinification trends, like in the Atlantic, still require at least a few decades, and the models differ by more than 50 yr, despite overall model agreement on the sign and magnitude of the trend (Figure S2). For the North and equatorial Pacific and Atlantic SSS emerges on time scales ranging from 20–50 yr.

For export production, chlorophyll, and SSS, emergence times for the RCP4.5 scenario can extend decades beyond those of RCP8.5. This indicates that in some regions, mitigation robustly delays the impacts of climate change.

#### 3.1.3 Local ToE

**Figure 3:**
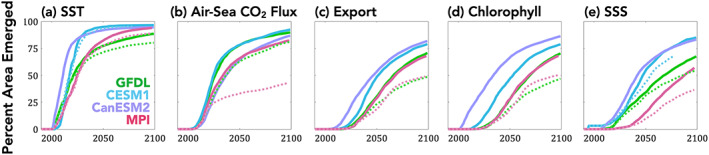
Pace of local emergence (percent of global ocean area emerged at each year) for RCP8.5 (solid lines) and RCP4.5 (dashed lines). Presentation of RCP4.5 local‐scale emergence estimates from CanESM2 is excluded for all variables and from CESM1 are excluded for the biogeochemical variables (air‐sea CO_2_ flux, export production, and chlorophyll) due to insufficient ensemble size.

**Figure 4:**
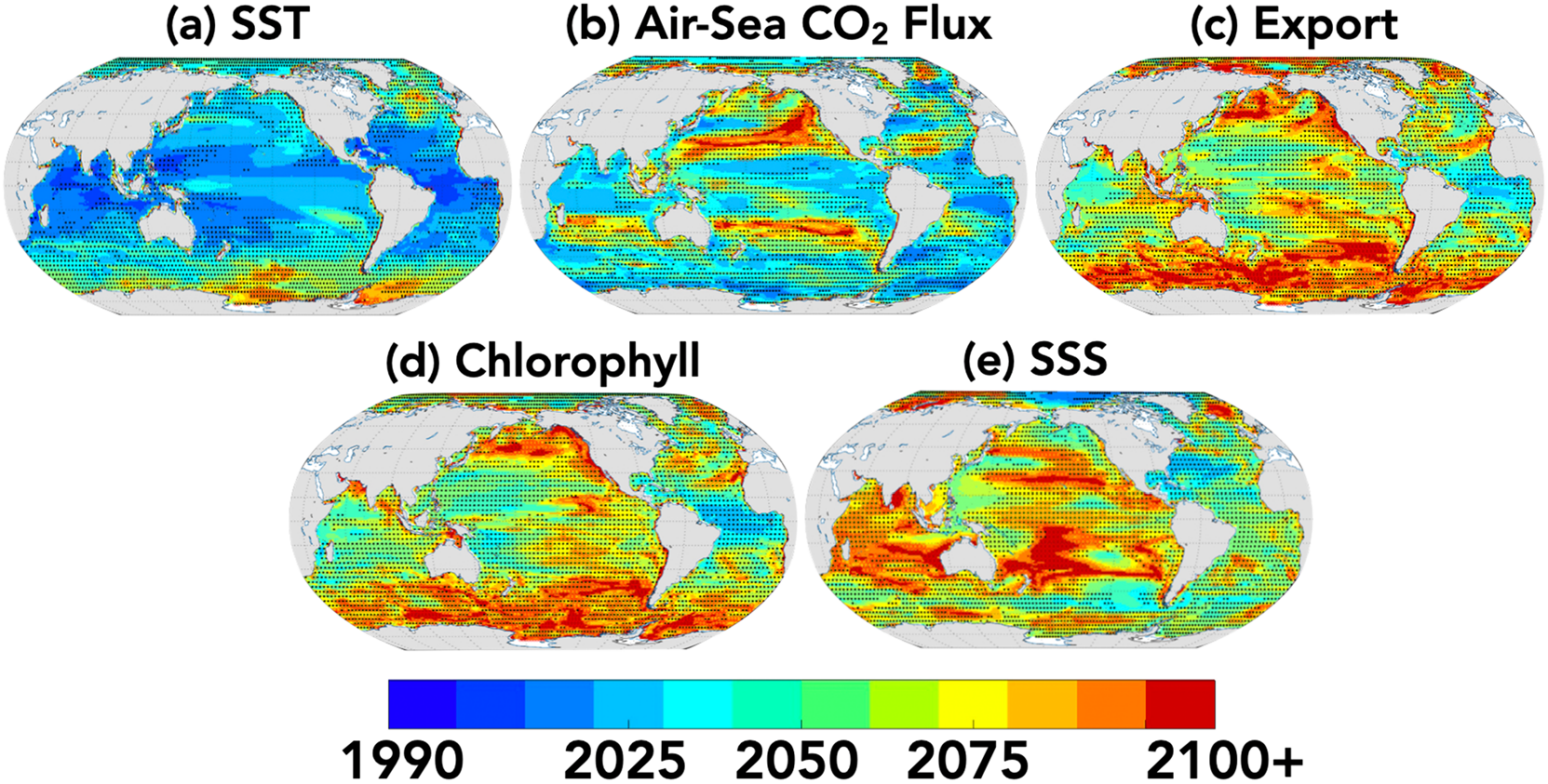
Maps of multimodel mean *Time of Emergence*. Black stippling indicates pixels where the spread (standard deviation) of the four LE's ToE is more than half the mean ToE, and red circles indicate pixels where more than one of the four LEs are nonemergent at the end of the century. For averaging purposes, Year 2100 was used when emergence does not occur for given LE.

**Figure 5:**
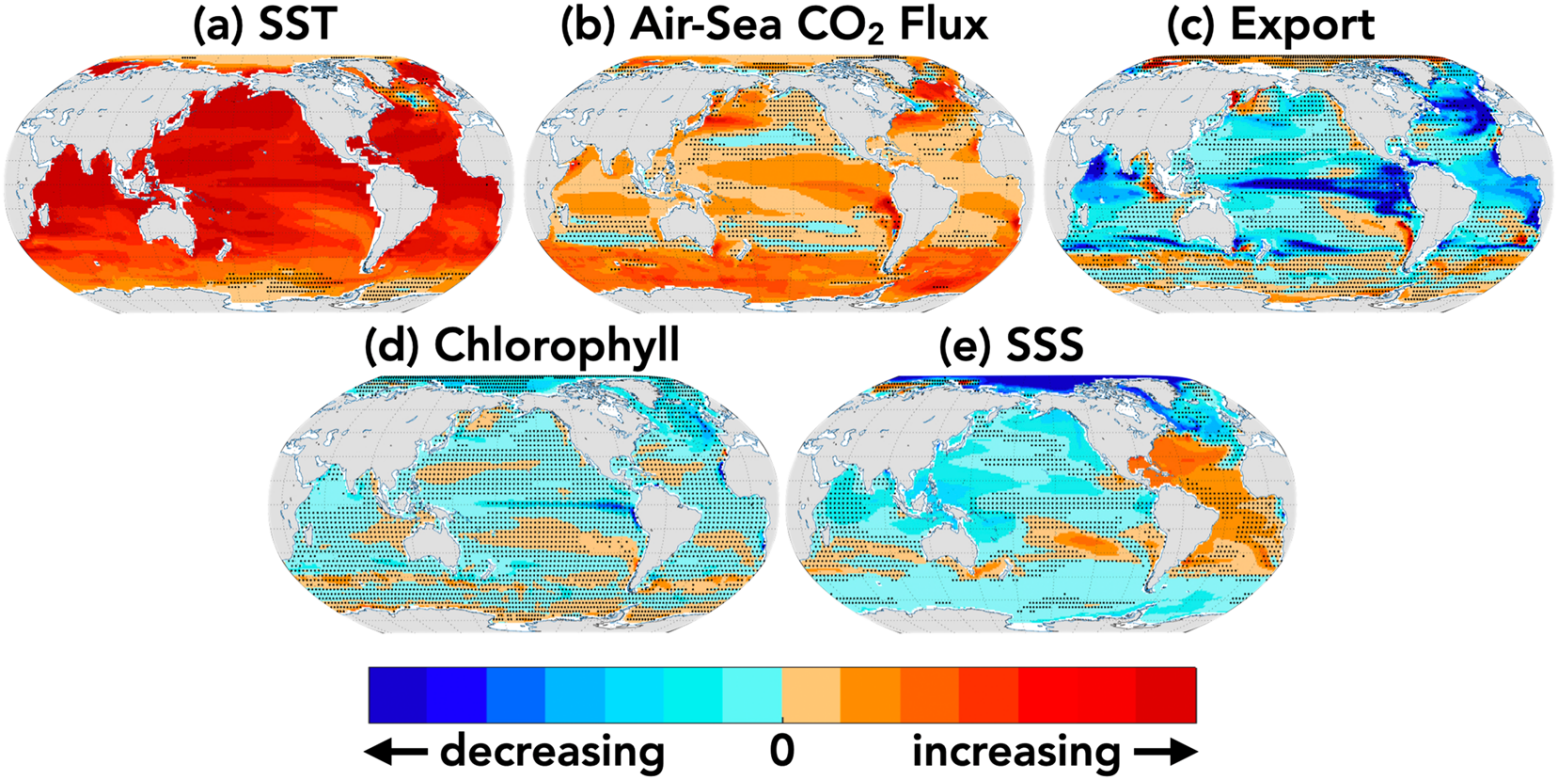
Maps of multimodel mean *signal* at Time of Emergence or Year 2100, whichever comes first. Black stippling over locations of where LEs disagree (mean signal of the four LEs is less than the spread of signals across models). The maximum value on the color bar for SST corresponds to 3°C/100 yr, for air‐sea CO_2_ flux to 53 g C/m^2^/yr/100 yr, for export production 15 g C/m^2^/yr/100 yr, for chlorophyll to 0.7 mg Chl/m^3^/100 yr, and for SSS to 1.7 psu/100 yr.

Now we consider emergence and signals at the local scale (Figures 3, 4, 5). We first consider the cumulative area of emerged grid cells over time, relative to Year 1990 (Figure 3). The four ESMs agree within approximately a decade upon the pace or rate of local emergence, with the exception of slowed emergence at end of century for ESM2M's SST (Figure 3a) and for chlorophyll and SSS for ESM 2M and MPI‐ESM‐LR (Figures 3d and 3e). Slowed emergence at end of century for ESM2M's SST field is a consequence of the aforementioned nonemerging Southern Ocean, due to the forced signal of weak cooling in this region (Figure S5; Manabe et al., 1990).

Air‐sea CO_2_ fluxes show strong agreement between the LEs not just for global and regional integrals (Figure 2) but also locally (Figure 3b) and spatially (Figure 4b). All LEs share the common feature of nonemergence in the Ekman convergence regions of the subtropical gyres (Figures 4b and 5b), as previously shown in McKinley et al. (2016) and Schlunegger et al. (2019) for CESM1 and ESM2M, respectively. Nonemergence of annual trends arises from the superposition of opposing seasonal trends, specifically enhanced summertime outgassing and enhanced wintertime uptake (Schlunegger et al., 2019).

Since ToE is a threshold‐based metric, averted emergence due to mitigation can be nonlinear. This is exemplified by mitigation resulting in a ~50% reduction in local emergence of air‐sea CO_2_ for the MPI‐ESM‐LR (Figure 3b). This occurs because in MPI‐ESM‐LR air‐sea CO_2_ fluxes in the subtropics and large regions of the Southern Ocean and North Pacific do not emerge until midcentury under RCP8.5 forcing, allowing for the effects of mitigation (RCP4.5) to delay emergence time scales (Figures S6d and S6f). This is in contrast to the ESM2M, for which mitigation has a small impact (~10%) on local ToE delays, because emergence occurs prior to the impacts of mitigation arising (Figures S6a and S6e).

In contrast, export production does not show agreement upon the locations of local emergence (Figures 4c and S6), despite showing agreement across the LEs in pace of local emergence (Figure 3c). The four ESMs also do not agree on the magnitude or direction of the export production change that emerge (Figures 5c and S6). For example, in the equatorial Pacific cold tongue region, ESM2M has a mixture of weak and nonemergent negative and positive trends, CESM1 has positive trends, and CanESM2 and MPI‐ESM‐LR have negative trends (Figure S7). Similarly, the Southern Ocean has divergent trends, with declining export for ESM2M, increasing export for CanESM2 and nonemergent local trends for MPI‐ESM‐LR and CESM1. Chlorophyll, like export, exhibits long time scales of local emergence, and significant model disagreement upon the timing of emergence, which localities emerge, and with what signal magnitude and direction (Figures 4, 5, and S8).

For SSS, the four ESMs agree on the underlying features of the signal. This includes salinification of the tropical and subtropical Atlantic and southern subtropical gyre of the Pacific Ocean, a freshening of the Arctic and North Atlantic, and weak freshening of the equatorial and North Pacific and the Indian and Southern Oceans (Figures 5e and S9). Despite agreement on the signal's spatial pattern, the LEs do not agree on local ToE (Figures 3e and 4e) as a consequence of the different signal magnitudes (larger for CanESM2 and MPI‐ESM‐LR) and differing noise (Figure S9).

### 3.2 SNRs for Anthropogenic Trends Over Observational Period

**Figure 6:**
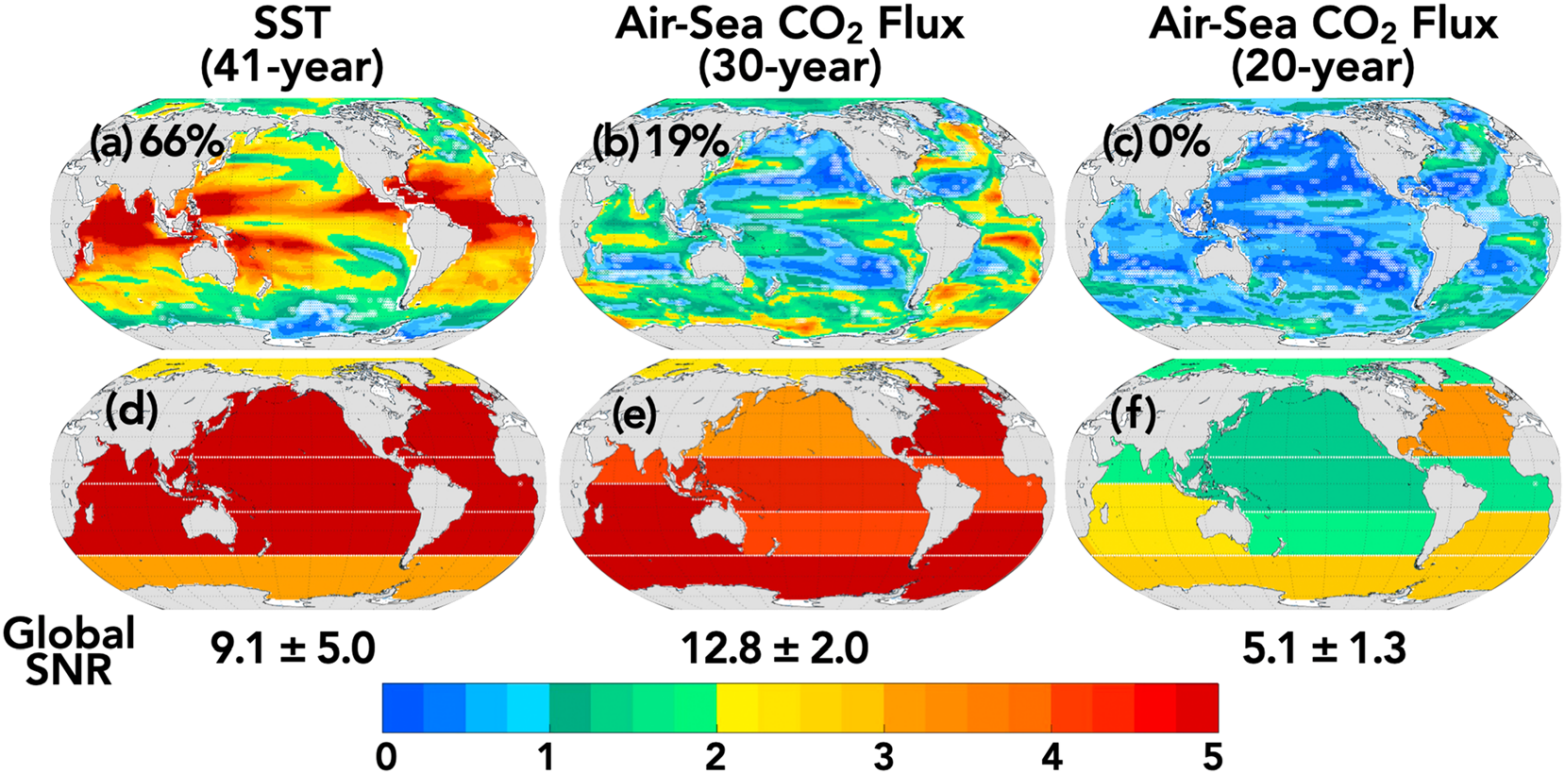
Maps of local (top row, a–c) and regional (bottom row, d–f) signal‐to‐noise ratio for the multi‐LE mean. White hatching indicates locations of where LEs disagree (where the multi‐LE mean SNR is less than the standard deviation of SNRs across the four models). Number of years over which the SNR ratio is estimated is given in parentheses. For SST, 41 yr trends for 1979–2019, and for air‐sea CO_2_ flux, 30 yr trends for 1990–2019 and 20 yr trends for 1990–2009. The multi‐LE mean global SNR ratio and the standard deviation across the multi‐LEs is given below the maps for each variable. The percent of ocean area with SNR > 2 is shown on upper left corner of Maps a–c.

**Figure 7:**
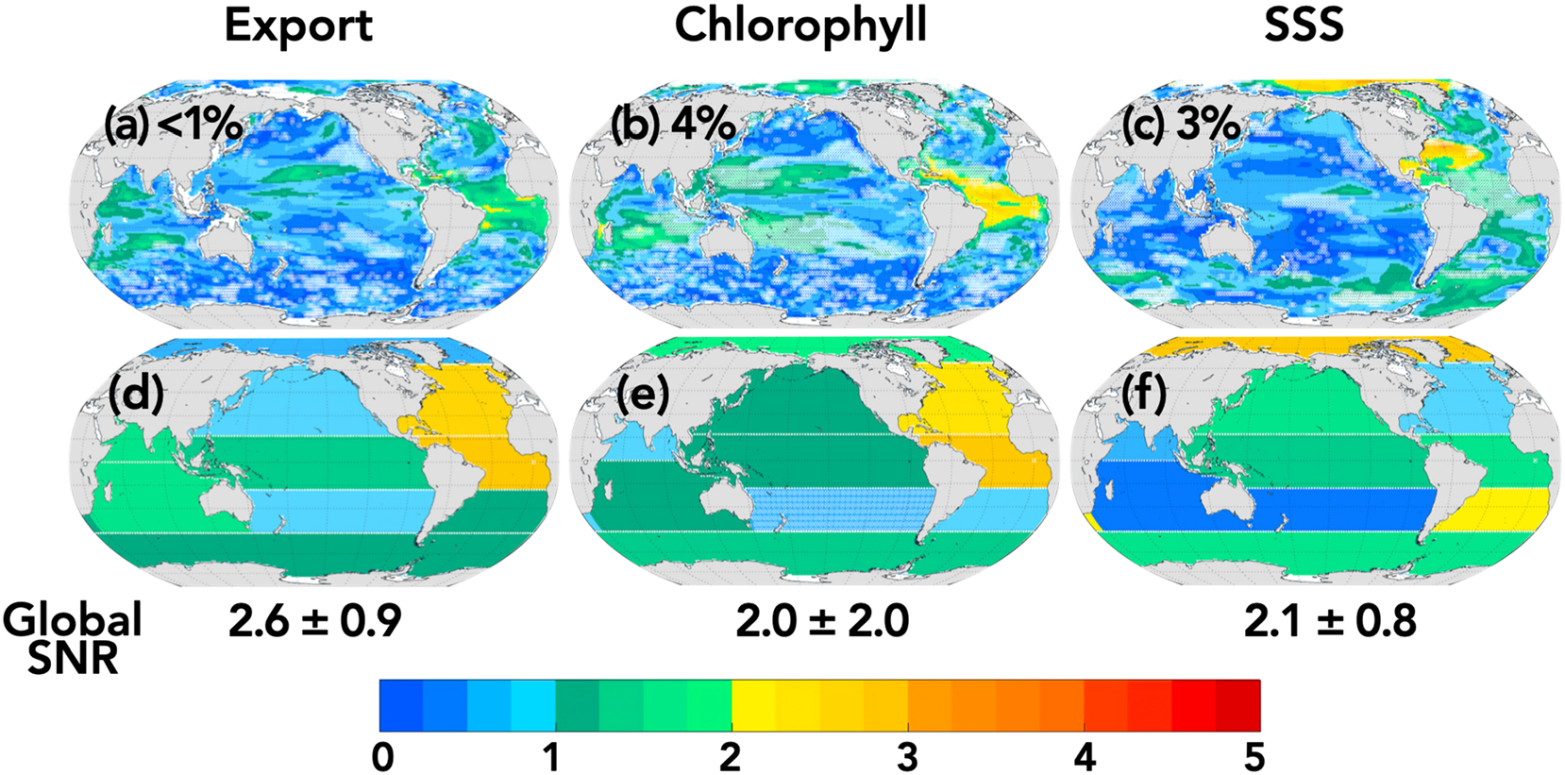
Maps of local and regional signal‐to‐noise ratios for the multi‐LE mean. White hatching indicates locations of where LEs disagree (where the multi‐LE mean SNR is less than the standard deviation of SNRs across the four models). For export production and chlorophyll the SNR is given for the 30 yr period 1998–2027. For sea surface salinity the SNR is given for the 30 yr period 2000–2029. The multi‐LE mean global SNR ratio and the standard deviation across the multi‐LEs is given below the maps for each variable. The percent of ocean area with SNR > 2 is shown on the upper left corner of Maps a–c.

Considering ToE from the same start date (1990) across variables is necessary for scientific and mechanistic interpretation of detectability and impact time scales across the spectrum of variables. However, in order to directly aid interpretation of the observational record, we include SNRs for the multimodel mean (e.g., Equation 4) over relevant observational time periods (Figures 6 and 7). A SNR > 1, 2, or 3 signifies emergence of an anthropogenic trend with 67%, 95%, and 99% confidence, respectively. As noted in the methods, we define a trend as emergent when the SNR > 2.

The first full year of globally resolved satellite estimates of SSTs was 1979, and therefore we provide the SNR for SST over the 41 yr period 1979 to 2019 (Figures 6a and 6d). Most of the available data‐based products for air‐sea CO_2_ fluxes (e.g., Rödenbeck et al., 2015) start on or before 1990, and so for air‐sea CO_2_ fluxes we provide SNRs for the 20 yr period, 1990–2009, used in the RECCAP (Wanninkhof et al., 2013) project and over the 30 yr period 1990–2019 which characterizes the duration of the effective carbon observing system (Figures 6b, 6c, 6e, and 6f).

SST is emergent for the globally integrated signal, for all regionally integrated signals, and locally for 66% of the global area (Figures 6a and 6d). This is consistent with the scientific consensus that global and regional warming of the upper ocean from midcentury to present is attributable to anthropogenic forcing (Bindoff et al., 2013). For the current 30 yr time period, 1990–2019, globally and regionally integrated air‐sea CO_2_ fluxes are emergent, with 19% of global area having emergent local trends (Figures 6b and 6e). This is in contrast to the 20 yr period 1990–2009, the RECAP period, for which 0% of grid cells and less than half of the regions are emergent (Figures 6c and 6f).

Global ocean color measurements span the 22 yr period 1998 to 2019; however, we show there is no emergence for surface chlorophyll concentrations or the export of organic carbon at the local, regional, or global scale (Figure S13). Looking to the near future, to the 30 yr period that will be available this decade, the four ESMs agree on the emergence of anthropogenic trends in the equatorial Atlantic and near‐emergent trends in the North Atlantic (Figures 7 and S15). The ESMs with high climate sensitivity (CanESM2 and CESM1) have stronger local, regional, and global emergence of export and surface chlorophyll (Figures S14 and S15).

For SSS, the Year 2000 marks the beginning of the ARGO program, the first continuous, near‐global salinity observing system (Durack et al., 2016); however, we show no emergence for regional or local trends over the 20 yr period 2000–2019 (Figure S13). Looking to the future again, to the 30 yr period 2000–2029, emergent trends appear in the South Atlantic, and the SNR approaches 2 for the equatorial Atlantic, North and equatorial Pacific, and Southern Oceans.

This indicates that approximately a decade more of observations of ocean color and SSS could result in the detection of regionally emergent anthropogenic trends. Furthermore, the LE mean global signals for these three fields become emergent during the 30 yr observational window considered.

### 3.3 Partitioning Uncertainty in Projections

**Figure 8:**
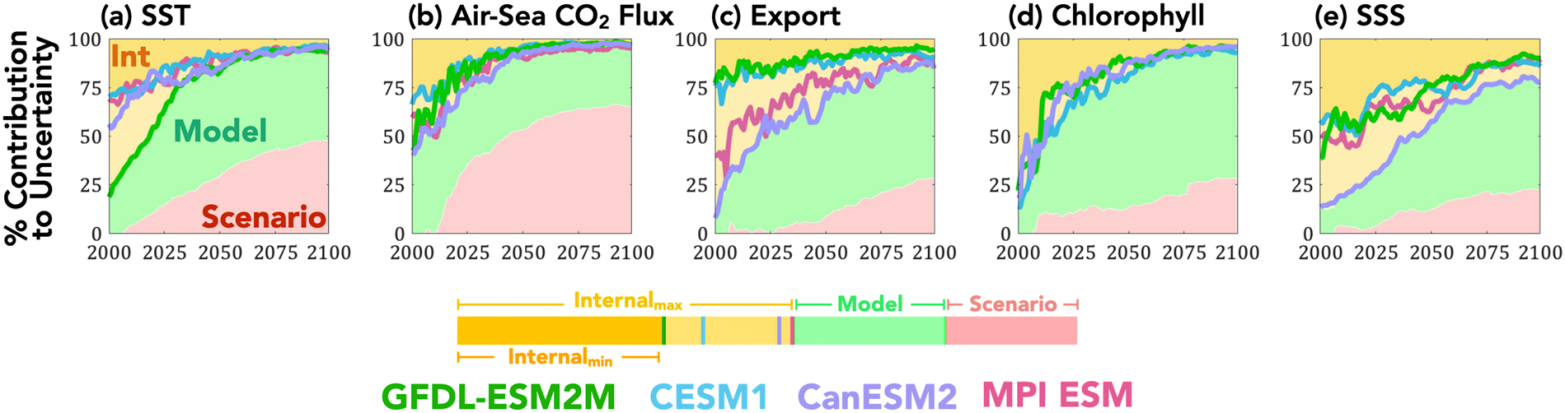
Partitioning uncertainty for globally averaged (a) SST, (b) air‐sea CO_2_ flux, (c) export production, (d) chlorophyll, and (e) SSS, for scenario uncertainty (red, RCP4.5 vs. RCP8.5), model uncertainty (green shading), and internal variability (yellow shading). The contribution of internal variability from each ESM (*U*_*IV,m*_) is given by the colored lines, with color codes as in previous figures. The boundary between light yellow and green is determined by maximum contribution from internal variability to total uncertainty, that is, the model with the largest internal variability at that point in time (*U*_*IV*_, Equation 10). The darker yellow shading occurs over the smallest contribution of internal variability between the four ESMs, indicating the minimum contribution of internal variability to total uncertainty. The structural uncertainty in internal variability contributions to projection uncertainty (Δ*UC*_*IV*_, Equation 13) or the difference between the maximum and minimum internal variability contributions is given in light yellow.

**Figure 9:**
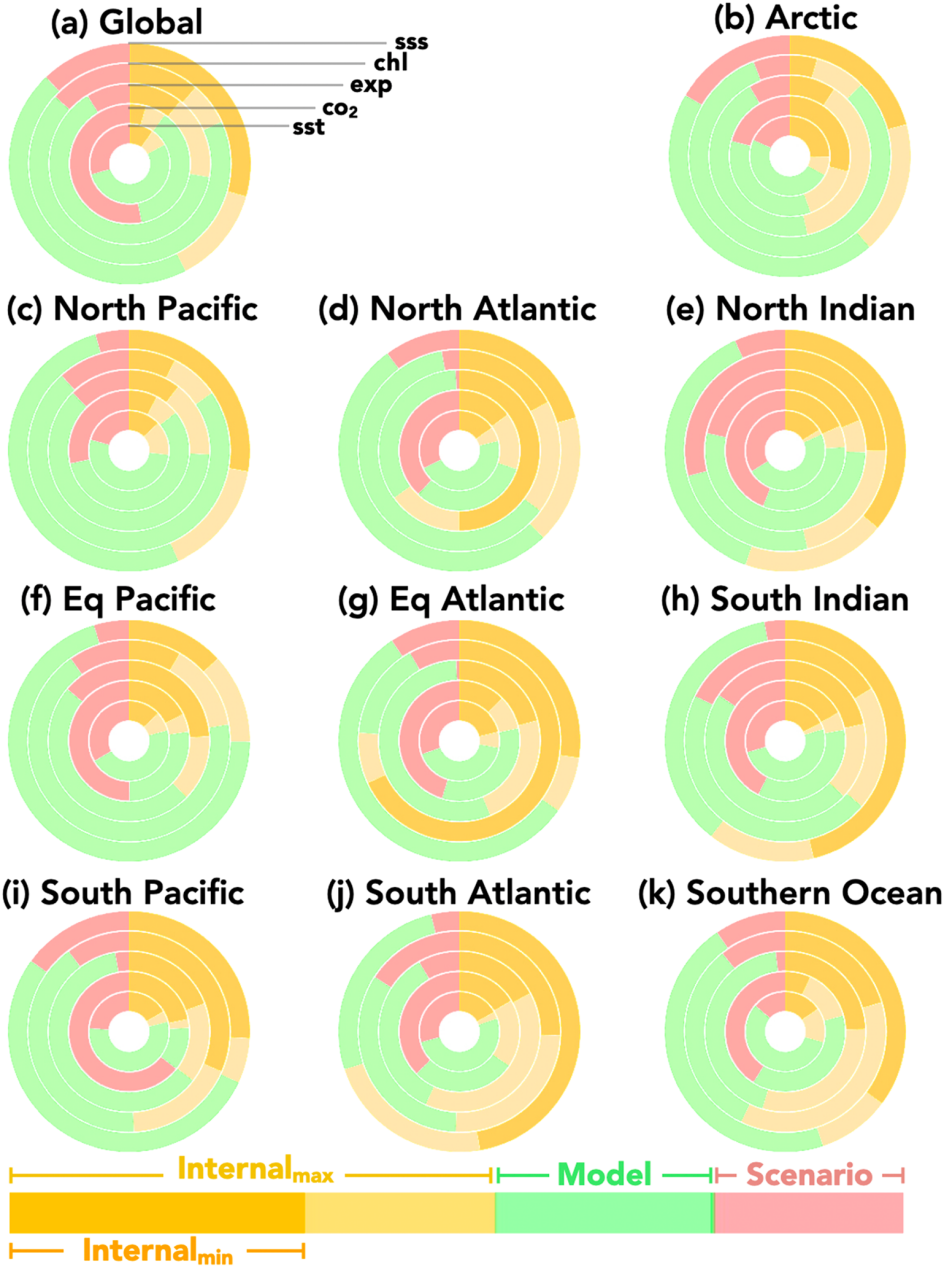
Partitioning global and regional uncertainty at Year 2050 for SST (innermost circle), air‐sea CO_2_ flux, export production (middle circle), chlorophyll, and SSS (outer circle). Scenario uncertainty (red, RCP4.5 vs. RCP8.5), model uncertainty (green shading), and internal variability (yellow shading). (a) A snapshot of globally integrated uncertainty shown in Figures 8a–8e at Year 2050. The boundary between light yellow and green is determined by maximum contribution from internal variability to total uncertainty, that is, the model with the largest internal variability at that point in time (*U*_*IV*_, Equation 10). The darker yellow shading occurs over the smallest contribution of internal variability between the four ESMs, indicating the minimum contribution of internal variability to total uncertainty. The structural uncertainty in internal variability contributions to projection uncertainty (Δ*UC*_*IV*_, Equation 13), or the difference between the maximum and minimum internal variability contributions, is given in light yellow.

We now turn to partitioning the sources of uncertainty in the LE projections (Figures 8, 9, and S16). Partitioning uncertainty in projected change is of interest because the different sources of uncertainty have different consequences or implications for future research and mitigation strategies. For example, if structural uncertainty is relatively large, scientific advances in modeling the Earth system would provide improved projection skill. Alternatively, if scenario uncertainty is large, this indicates that societal decisions are important for the given outcome. If natural internal variability uncertainty is large, this indicates change may not be discernable from background noise and nor would the impacts of differing societal decisions. Large internal variability uncertainty also implies that detecting the given response of the Earth system would require sustained observations and that organisms and systems may have high tolerance or resilience to change, as the envelope of variability to which they are adapted is large relative to the impact of the external forcing. The availability of LEs enables a robust estimate of the forced response, variability, and potential changes in variability for each model. Thereby, one can avoid making assumptions about the partitioning between forced response and internal variability as in the original methodology (Hawkins & Sutton, 2009). Equations 1–13 document our methodology for partitioning uncertainty.

At global scales, scenario and model uncertainty are both important for multidecadal projections of SST (Figure 8). Scenario uncertainty becomes increasingly dominate over time for air‐sea CO_2_ (Figure 8, consistent with Frölicher et al., 2016, and Lovenduski et al., 2016, respectively). For export production, surface chlorophyll concentrations, and SSS, model uncertainty dominates at the global scale and for most regions by midcentury (Figures 8 and 9).

With the use of multiple LEs, we have multiple estimates of the contribution of internal variability uncertainty to total uncertainty, which is given by the individual colored lines within the yellow “internal” sections of each figure and described by Equation 11. Previous methods used either the noise from a preindustrial control run of a single climate model or ESM, an average across single ensemble members from multiple ESMs, or the noise from a LE of a single ESM. Here we can take the novel approach of providing noise estimates from contemporary LEs from multiple ESMs. This provides an estimate of the uncertainty *of* the magnitude of internal variability uncertainty (Δ*UC*_*IV*_, Equation 13), which is represented by the spread of the four ESMs individual lines which bound the light yellow region in Figures 8 and 9.

Differences between the ESMs' estimates of uncertainty contributions from internal variability is most pronounced for export production, surface chlorophyll concentrations, and SSS, particularly at the regional scale, where internal variability is generally a larger contribution to total uncertainty. For example, in the Southern Ocean (Figure 9k), internal variability uncertainty is the dominate source of uncertainty (>50%), and differences between ESMs estimates of internal variability uncertainty exceed 25% even at midcentury. For the biological variables presented here, the dominance of internal variability for many regions, and the difference in estimates of internal variability provided by the ESMs, illustrates the importance of the estimation of natural internal variability for assessing and predicting change over the coming decades.

The multiple LEs demonstrate that the contributions of internal variability to projection uncertainty is model‐dependent and an important source of uncertainty for decades to come for some variables and many regions (Figure 9). However, not explicitly represented here is the uncertainty associated with the characterization of model and scenario uncertainty. Our methodology and the use of only four models and two scenarios potentially underestimates the contribution of these two sources of uncertainty, as inclusion of more models and strong‐mitigation scenario could only act to maintain or increase the range of projected anthropogenic changes. As noted, our selection of models does include end‐members (minimum and maximum) of the CMIP5 projections for end‐of‐century globally averaged changes in SST and net primary production (NPP; Bopp et al., 2013) and air‐sea CO_2_ fluxes (Arora et al., 2013). This indicates the model uncertainty in SST, air‐sea CO_2_ fluxes, biological export and chlorophyll (both strongly correlated to NPP), and SSS (for which the magnitude of anthropogenic perturbations scale with transient climate sensitivity; Durack et al., 2012) presented here is broadly representative of known model uncertainty. Beyond the known uncertainties derived from the range of possible emission scenarios and model representation of forced changes and internal variability are additional, currently unquantifiable uncertainties, which could also act to increase and repartition contributions from the sources of uncertainty to future projection uncertainty (e.g., Jones, 2000; Tebaldi & Knutti, 2007).

## 4 Conclusions

We have conducted a comprehensive multi‐LE analysis of ToE for a range of biogeochemically pertinent variables in the upper ocean, enabling us to provide a multifaceted and quantitative view of uncertainties associated with climate change projections with Earth system models. This was facilitated by the recent availability of a suite of four biogeochemically inclusive LE simulations with models from disparate international laboratories. Our interest here in particular is in carbon and carbon‐related variables of importance to climate, and in addition to our interest in quantifying projection uncertainty we have also discussed mechanistic attribution and implications for observing system design.

We wish to emphasize first the commonalities among the models, as consistent results across models can be expected to offer confidence in informing observing system design and duration. This was considered for the role of natural internal variability uncertainty for each ESM evaluated independently. We find the chronology of emergence to be consistent among the four ESMs evaluated in this study. Specifically, SST emerges first, followed by air‐sea CO_2_ fluxes, then export production, followed next by surface chlorophyll concentrations and then finally SSS. We interpret this chronology to reflect the time lag between the underlying drivers of the change (Schlunegger et al., 2019). Rising atmospheric CO_2_ warms the atmosphere and the surface ocean and induces a net positive “invasion flux” of CO_2_ into the ocean. Changes in ocean circulation subsequently and slowly result from surface heating and changes in surface freshwater fluxes. These changes in circulation, stratification, and/or transport eventually alter biological activity and export through model‐dependent pathways (e.g., Laufkötter et al., 2016). Additionally, the ESMs all demonstrate the power of large spatial footprints in reducing emergence time scales, implied by the finding that emergence times are generally shorter for globally integrated quantities than for regionally integrated quantities (Figure 2) and generally shorter for regional‐integrated quantities than for local or grid cell scale quantities (Figure 2 vs. Figures 3 and 4).

Our analysis considered the important question of how mitigation (manifested in divergent warming scenarios) impact emergence time scales. The ESMs broadly agree on which variables' ToE respond to moderate mitigation (with the exception of air‐sea CO_2_ fluxes). For variables that emerge rapidly, like SST, committed warming (due to past and unavoidable emissions) is sufficient to produce an emergent signal prior to impacts of climate mitigation efforts. However, for variables which emerge slowly, like export production, chlorophyll, and SSS, ToEs are scenario‐dependent and can be delayed by multiple decades with moderate climate mitigation efforts. The effects of mitigation on the emergence of local air‐sea CO_2_ fluxes presents an interesting case study of how modest (~10 yr) ToE differences between the ESMs under strong anthropogenic forcing can evolve into pronounced (60+ yr) ToE differences between the ESMs with moderate mitigation.

The global and regional partitioning of uncertainty presented in Figures 8 and 9 highlight an apparent paradox in that the scenario sensitivity of ToEs and scenario uncertainty in projection are inversely related. In other words, variables whose ToEs are insensitive to mitigation over time scales in the CMIP5 scenarios considered here (SST and air‐sea CO_2_ flux) have the largest future scenario uncertainty. However, these findings are in fact consistent, as variables which are sufficiently sensitive to emissions emerge early, prior to the impact of differential scenarios. For that case the choice of scenario does not alter ToE but does strongly influence the evolution of the signal over the 21st century (Figures 1, S3, and S4).

The analysis presented in Figures 6 and 7 indicate that the current observational record is long enough to identify global and regional anthropogenic trends in SST and air‐sea CO_2_ fluxes, the properties associated directly with rising CO_2_ concentrations and atmospheric temperature. For the attribution of local trends, however, the duration of observations is insufficient, particularly given the additional uncertainties associated with observations (like measurement error and gap filling) that we do not consider here. For air‐sea CO_2_ fluxes, the LEs agree that within the last decade, the duration of observational record has surpassed a critical threshold for regional emergence of anthropogenic trends.

For export production, surface chlorophyll, and SSS, properties indirectly associated with rising atmospheric CO_2_ concentrations, the observational record is likely insufficient for even global anthropogenic trends to be identified. However, in the coming decade, the LEs agree that regional trends in biological activity, export, and salinity could begin to emerge. These results indicate the high requirements for long‐term climate quality observing and the role of region‐based synthesis efforts toward variability assessment and anthropogenic trend detection. For SST and export production, the ToE is more robust across models than the magnitude or direction of the trend itself. In other words, for the models considered here the time scales over which anthropogenic signals emerge has more certainty than the characteristics of the underlying signal. Despite uncertainty in *what* signal will emerge, there is agreement in how long, at minimum, we must monitor in order to detect such a signal.

Confidence in the results is elevated when the different LEs agree among themselves. However, future research will have to assess the credibility of the models' trends and variability by comparing to observations whenever possible. Currently, the observational record is insufficient to characterize decadal and multidecadal variability for many ocean biogeochemical properties and processes. However, ToE research which leveraged atmospheric reanalysis products to estimate historical variability in atmospheric circulation has shown that systematic model biases in variability exist, such that—when corrected—ToE occurs systematically earlier or later as compared to purely model‐simulated data (Lehner et al., 2017; Santer et al., 2007). The significant disagreement between the magnitudes of different ESMs global and regional internal variability presented in Figures 8 and 9 suggests this could also be the case for ocean biogeochemical variables. Without a collection of LEs, it would be difficult to determine the extent to which an estimate of model uncertainty is contaminated with internal variability uncertainty. The degree to which LE initialization procedures (microperturbations vs. macroperturbations) influences the characteristics and magnitude of the internal variability demonstrated by the LEs is still an open question. The significant model/structural uncertainty and natural internal variability uncertainty underscores the importance of continued observational records to assess models and provide the best‐possible estimate of the imminent vulnerability of marine ecosystems under the combined influence of anthropogenic forcing and internal variability.

## Conflict of Interest

There are no competing interests to declare.

## Supporting information

Supporting Information S1

## Data Availability

The Multimodel Large Ensemble output used in this study is publicly available through Globus. The data archive can be accessed through https://poseidon.princeton.edu, and the documentation and description of the archive can be found at https://www.sarahschlunegger.com/large-ensemble-archive.
