# Supplementary material for: Time of Emergence and Large Ensemble Intercomparison for Ocean Biogeochemical Trends
Source: Global Biogeochem Cycles. 2020 Aug 23;34(8):e2019GB006453. doi: 10.1029/2019GB006453 (PMC7507776; doi:10.1029/2019GB006453)
Supplement: Supplementary file 1 — Supporting Information S1 [file GBC-34-e2019GB006453-s001.pdf]

AGU PUBLICATIONS

Global Biogeochemical Cycle

Supporting Information for

Time of Emergence and Large Ensemble Intercomparison For Ocean Biogeochemical Trends

Sarah Schlunegger[1*], Keith B. Rodgers[2,3], Jorge L. Sarmiento[1], Tatiana Ilyina[4], John Dunne[5], Yohei Takano[4,6], James Christian[7], Matthew C. Long[8], Thomas L. Frölicher[9,10], Richard Slater[1], Flavio Lehner[8]

[1] Program in Atmospheric and Oceanic Sciences, Princeton University, Princeton, New Jersey, USA
[2] Center for Climate Physics, Institute for Basic Science, Busan, South Korea
[3] Pusan National University, Busan, South Korea
[4] Max Plank Institute for Meteorology, Hamburg Germany
[5] NOAA Geophysical Fluid Dynamics Laboratory, Princeton, New Jersey, USA
[6] Los Alamos National Laboratory, Los Alamos, USA
[7] Canadian Center for Climate Modeling and Analysis, Victoria, British Columbia, Canada
[8] National Center for Atmospheric Research, Boulder, USA
[9] Climate and Environmental Physics, Physics Institute, University of Bern, Switzerland
[10] Oeschger Centre for Climate Change Research, University of Bern, Switzerland

Contents of this file

Figures S1 to S16

Supporting Figures

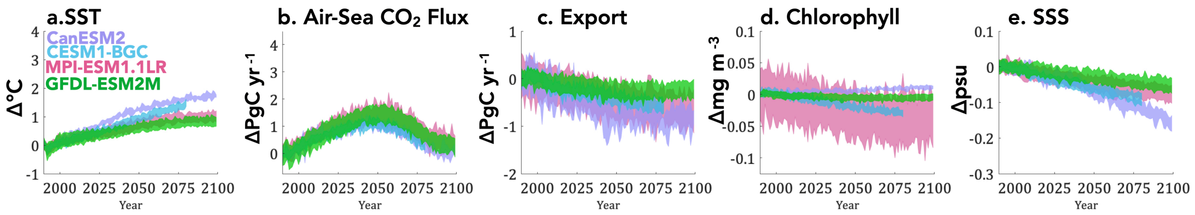

Figure S1. Global annual changes for **RCP4.5** ensembles relative to year 1990 for a. global mean sea surface temperature (SST), b. globally integrated air-sea CO_2 Flux, c. POC Export, d. surface chlorophyll and e. sea surface salinity (SSS). POC is particulate organic carbon.

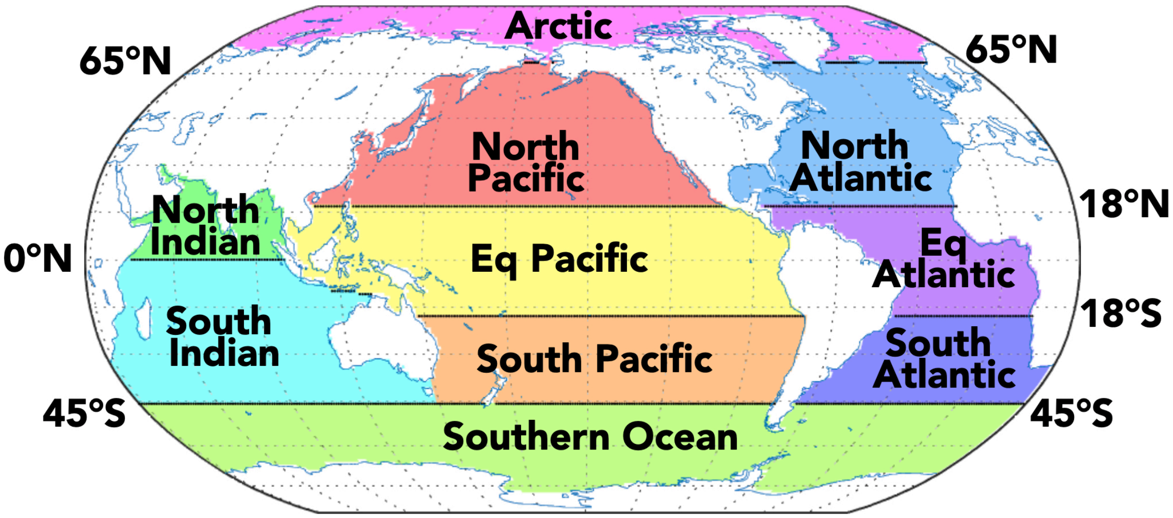

Figure S2. Map of regional domains used throughout this study.

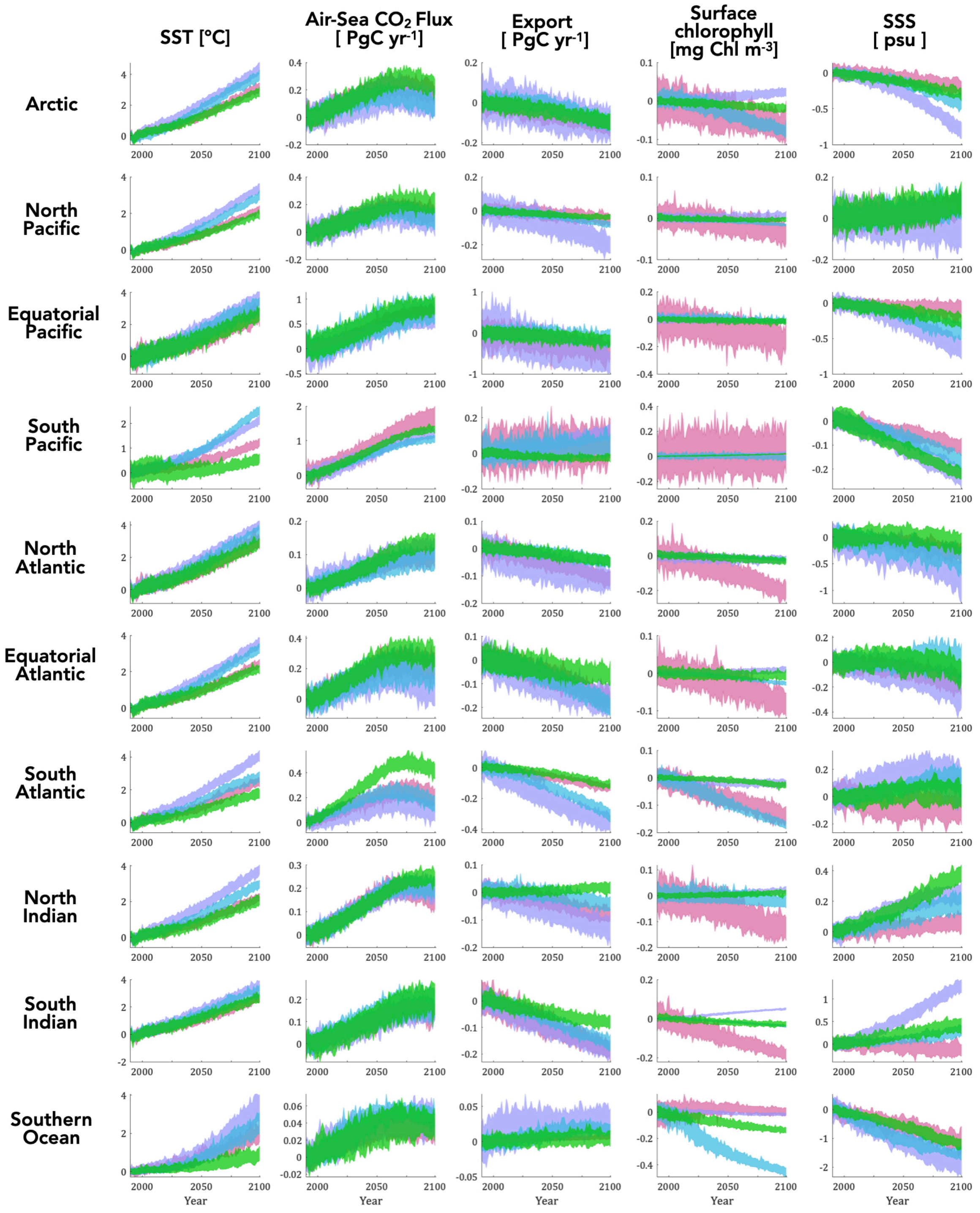

Figure S3. Regional time series of changes relative to year 1990 for **RCP8.5** scenario. Legend same as Figure 1 & S1 (GFDL green, CESM blue, CanESM2 purple, MPI pink). Variable indicated at top of each column.

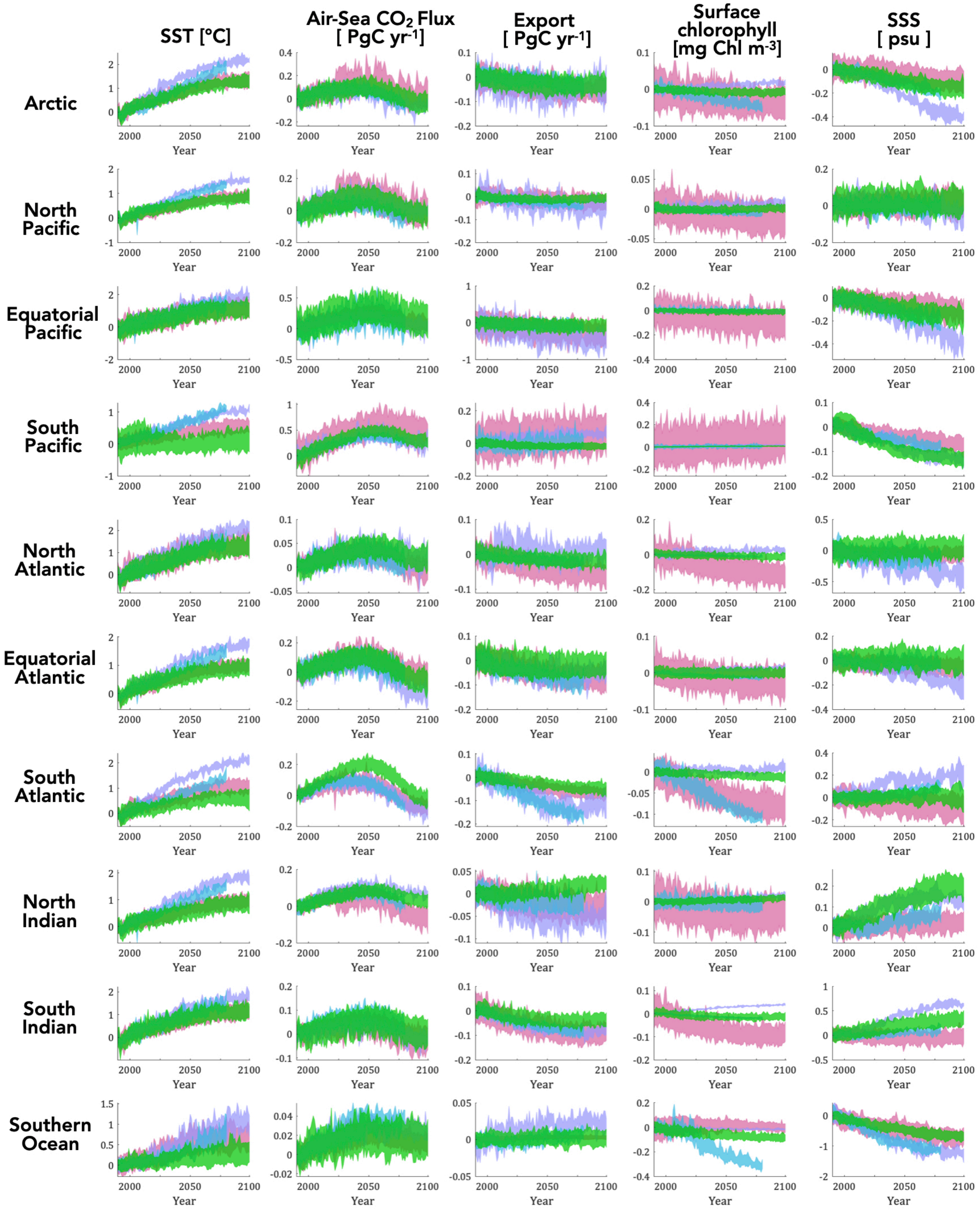

Figure S4. Regional time series of changes relative to year 1990 for **RCP4.5** scenario. Legend same as Figure 1 & S1 (GFDL green, CESM blue, CanESM2 purple, MPI pink). Variable indicated at top of each column.

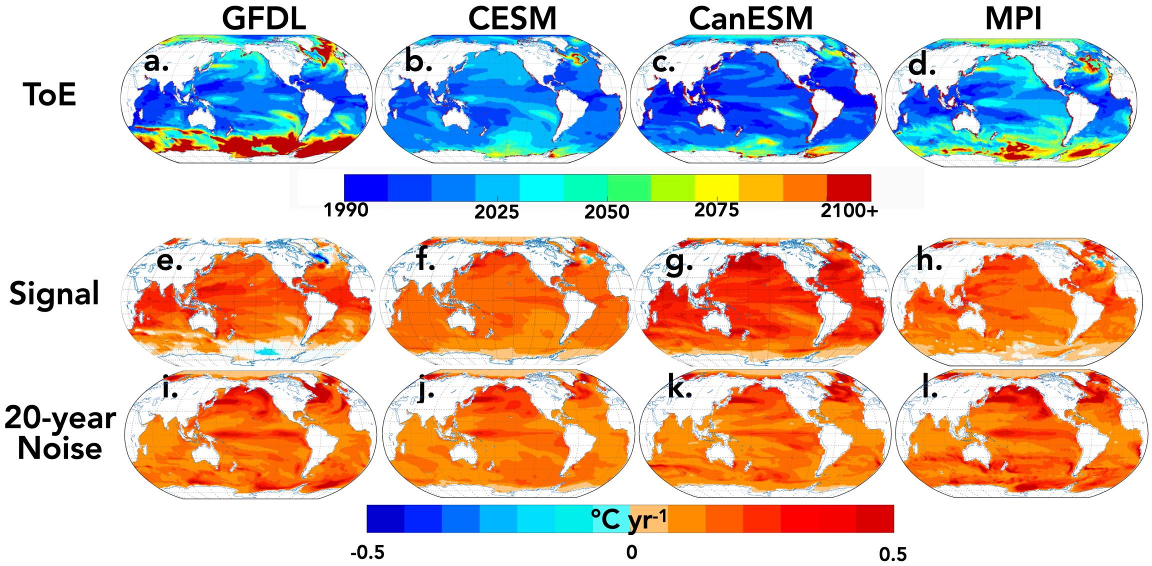

Figure S5. Maps of **sea surface temperature** emergence (a-d) and trends at time ToE (e-h) and the magnitude of 20-year variability (i-l) for the given models. Units of a-d are years. Units of e-l are degrees C per year. The trend is given at the Time of Emergence. White hatching on pixels with non-emergent trends are year 2100.

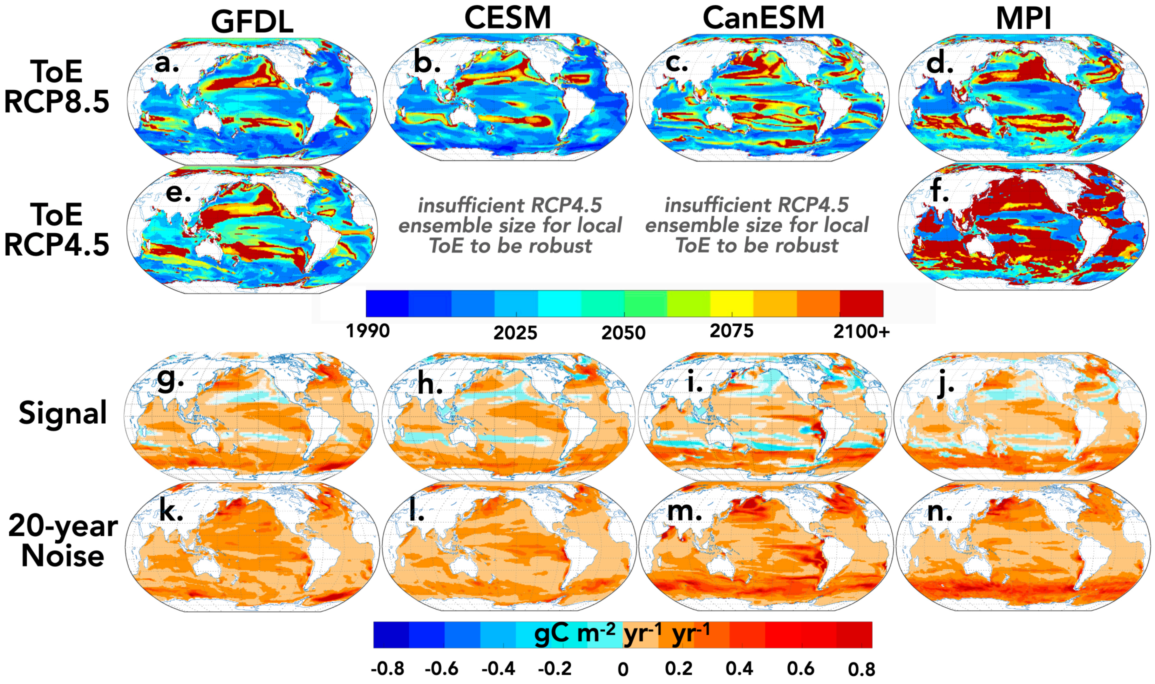

Figure S6. Maps of **air-sea CO_2 flux** emergence for RCP8.5 (a-d), RCP4.5 (e-f) and RCP8.5 trends at time ToE (e-h) and the magnitude of 20-year variability (i-l) for the given models. Units of e-l are gC meter^{-2} year^{-1} year^{-1}. The trend is given at the Time of Emergence. White hatching on pixels with non-emergent trends at year 2100. Insufficient ensemble size for RCP4.5 CESM Medium Ensemble and CanESM2 small ensemble.

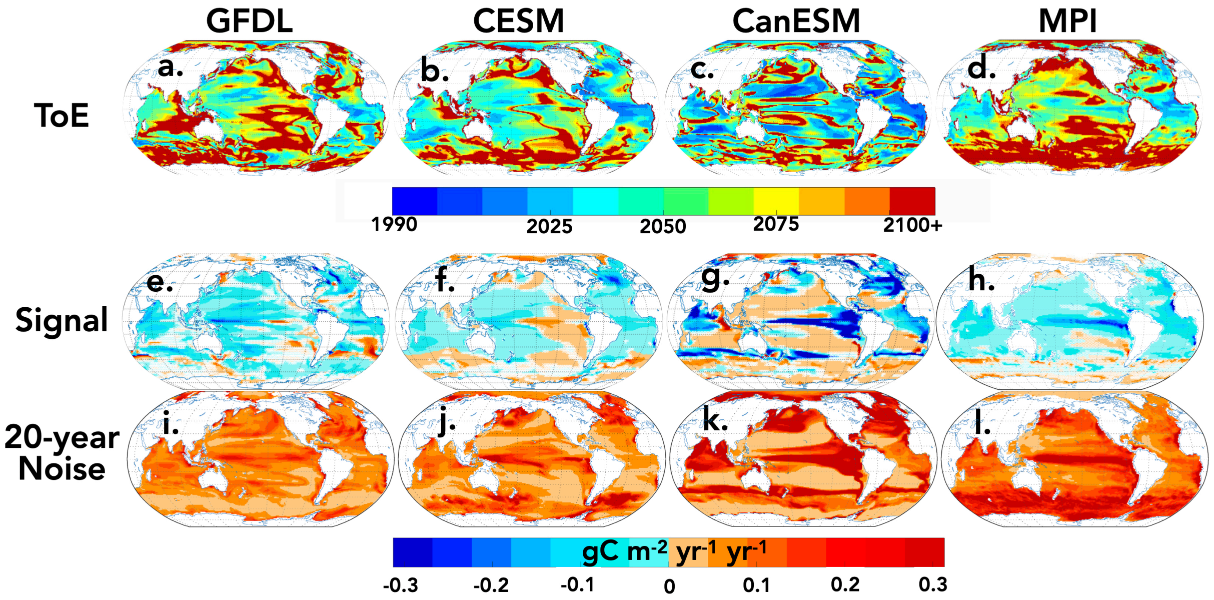

Figure S7. Maps of **export production** emergence (a-d) and trends at time ToE (e-h) and the magnitude of 20-year variability (i-l) for the given models. Units of a-d are years. Units of e-l are gC meter^{-2} year^{-1} year^{-1}. The trend is given at the Time of Emergence. White hatching on pixels with non-emergent trends are year 2100.

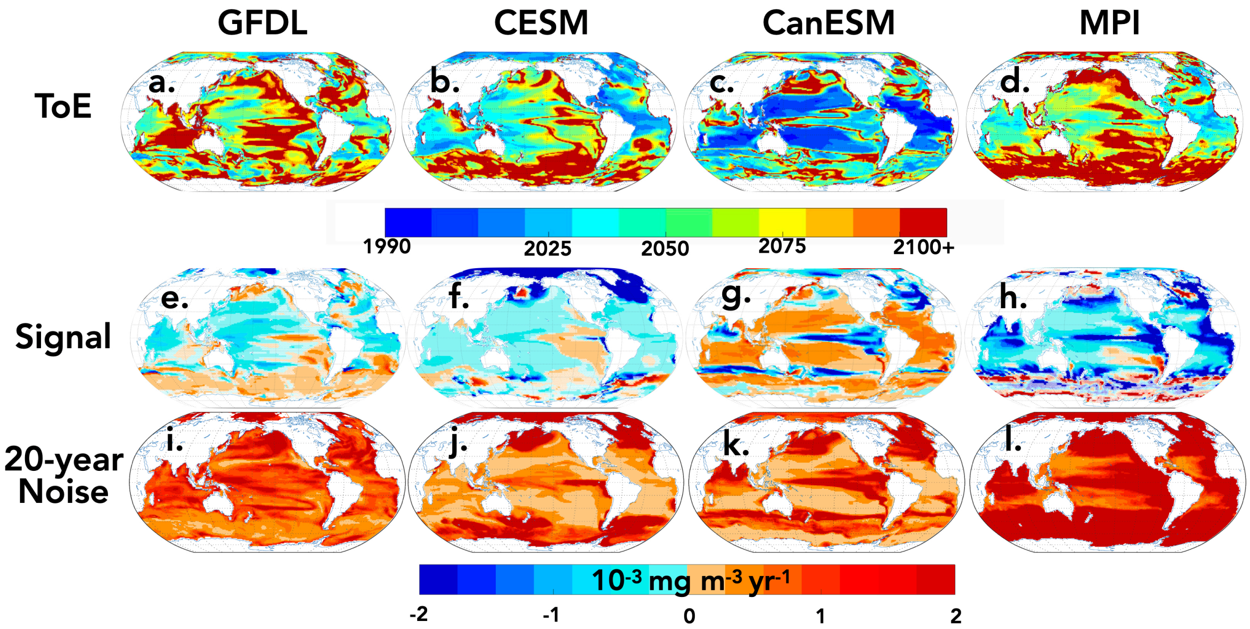

Figure S8. Maps of **surface chlorophyll** emergence (a-d) and trends at time ToE (e-h) and the magnitude of 20-year variability (i-l) for the given models. Units of a-d are years. Units of e-l are 10^{-3} mg m^{-3} per year. The trend is given at the Time of Emergence. White hatching on pixels with non-emergent trends at year 2100.

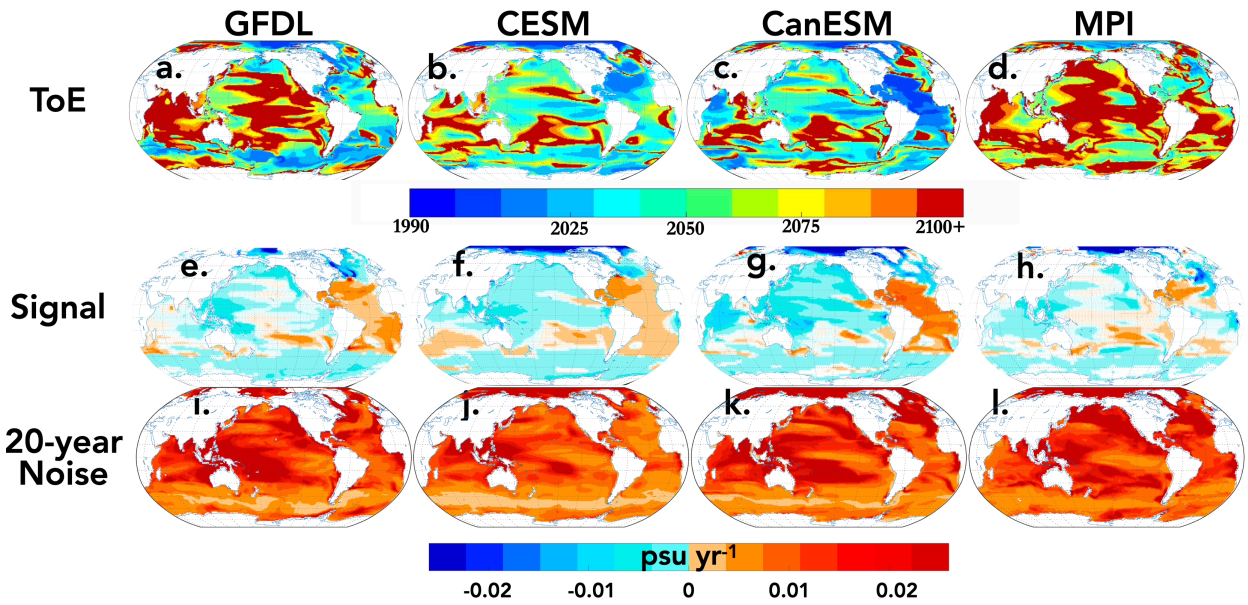

Figure S9. Maps of **sea surface salinity** emergence (a-d) and trends at time ToE (e-h) and the magnitude of 20-year variability (i-l) for the given models. Units of e-l are practical salinity units (PSU) per year. The trend is given at the Time of Emergence. White hatching on pixels with non-emergent trends are year 2100.

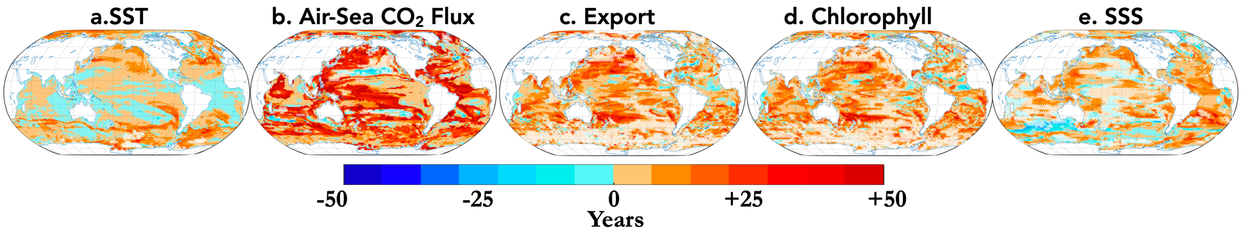

Figure S10. Maps of **difference in years between RCP4.5 and RCP8.5** multi-LE **mean ToE**. White hatching over locations of where mean difference (RCP4.5-RCP8.5) is less than 10% of the mean RCP8.5 TOE. For averaging purposes, year 2100 was used when emergence was not achieved for a given LE. For presentation of RCP4.5 local scale ToEs, CanESM2 LE excluded for all variables and CESM1-BGC excluded from air-sea CO_2 flux, export and chlorophyll due to insufficient RCP4.5 ensemble size.

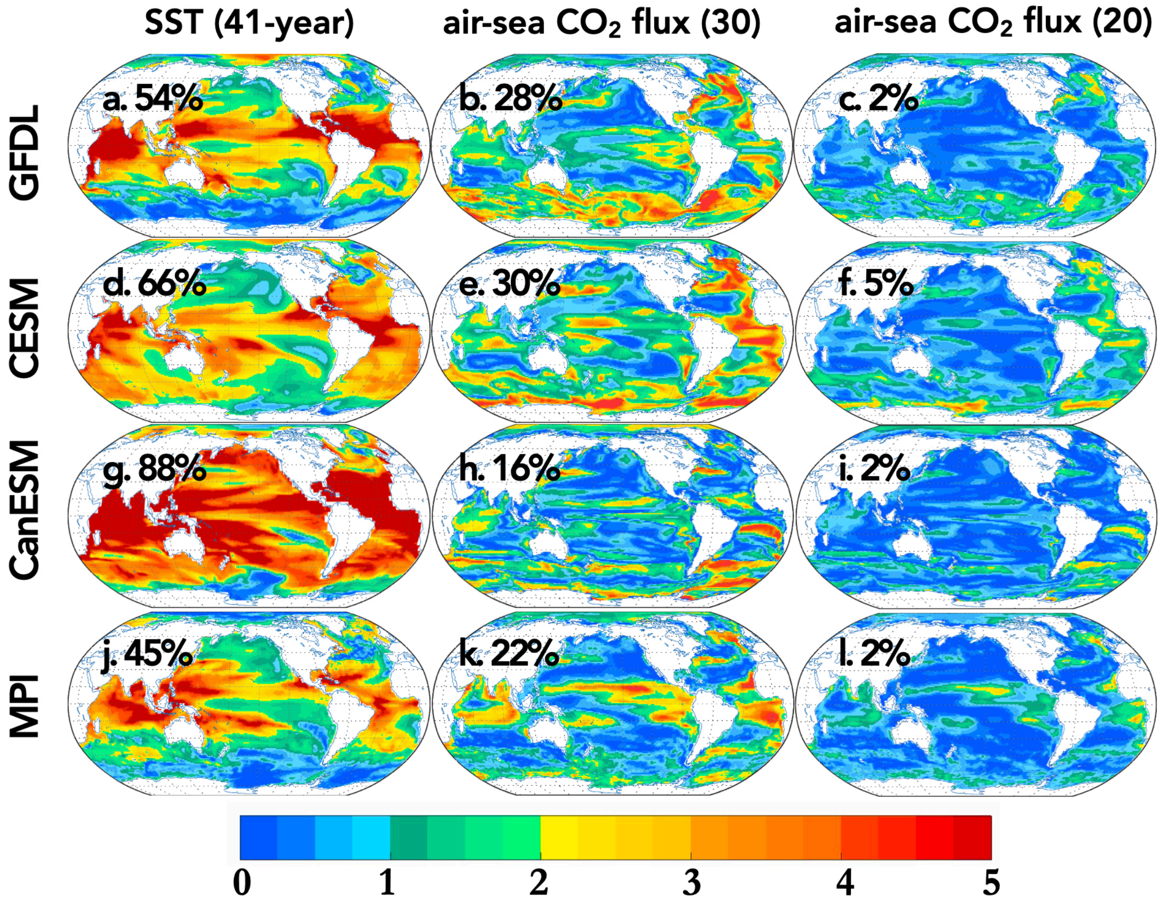

Figure S11. Maps of **Signal-to-Noise Ratio** for the four Large Ensembles, SST and Air-Sea CO_2 Flux. Number of years over which SNR ratio is estimated is given in parentheses. For SST, 41-year trends for 1979-2019, for air-sea CO_2 flux 30-year trends for 1990-2019 and 20-year trends for 1990-2009. The percent of ocean area with SNR > 2 shown on upper right corner of each map.

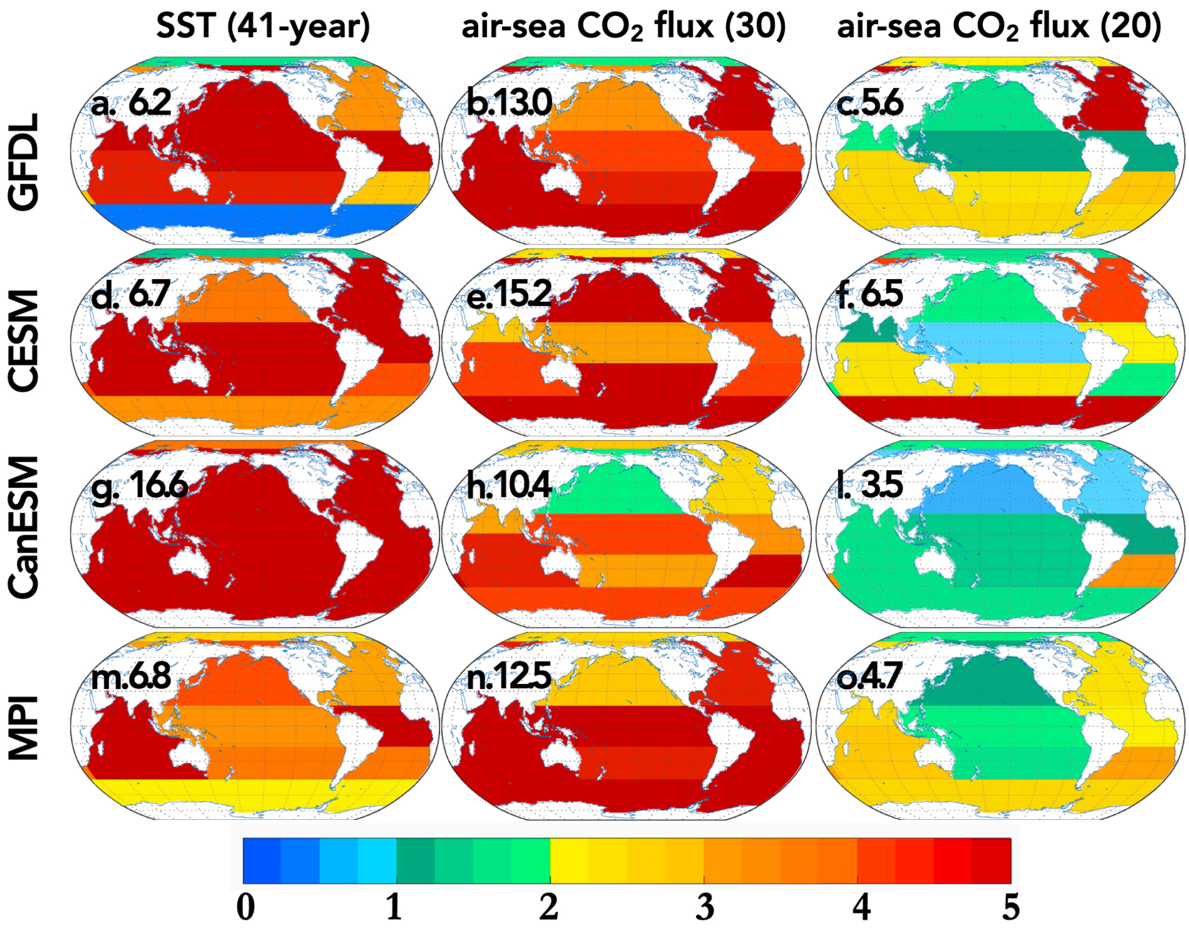

Figure S12. Maps of **regional Signal-to-Noise Ratio** for the 4 Large Ensembles, SST and Air-Sea CO_2 Flux.. Number of years over which SNR ratio is estimated is given in parentheses. For SST, 41-year trends for 1979-2019, for air-sea CO_2 flux 30-year trends for 1990-2019 and 20-year trends for 1990-2009. Each LEs Global SNR ratio given on upper left corner of map for each variable.

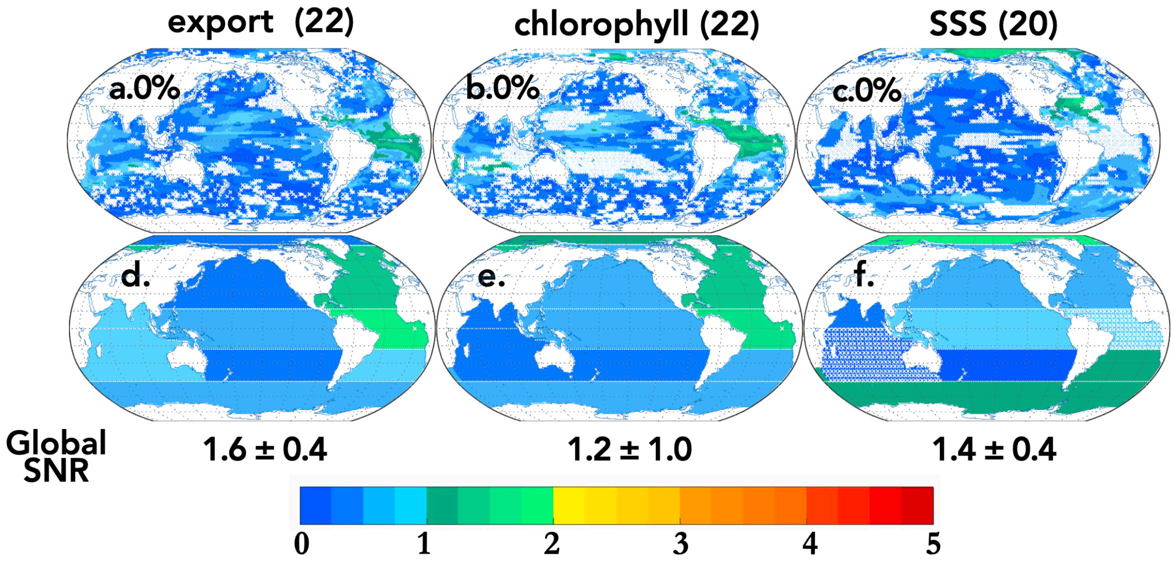

Figure S13. Maps of **Signal-to-Noise Ratios** for Export, Chlorophyll and SSS. White hatching over locations of where LEs disagree [where the multi-LE mean SNR is less than the standard deviation of SNRs across the four models]. **Number of years that SNR ratio is taken given in parenthesis**. The end-year of the trend for each variable is 2019. For export and surface chlorophyll, 22-year trends for 1998-2019 and for SSS, 20-year trends from 2000-2019. The LE mean Global SNR ratio ± the standard deviation across the LEs given below the maps of each variable. The percent of ocean area with SNR > 2 shown on upper right corner of each map.

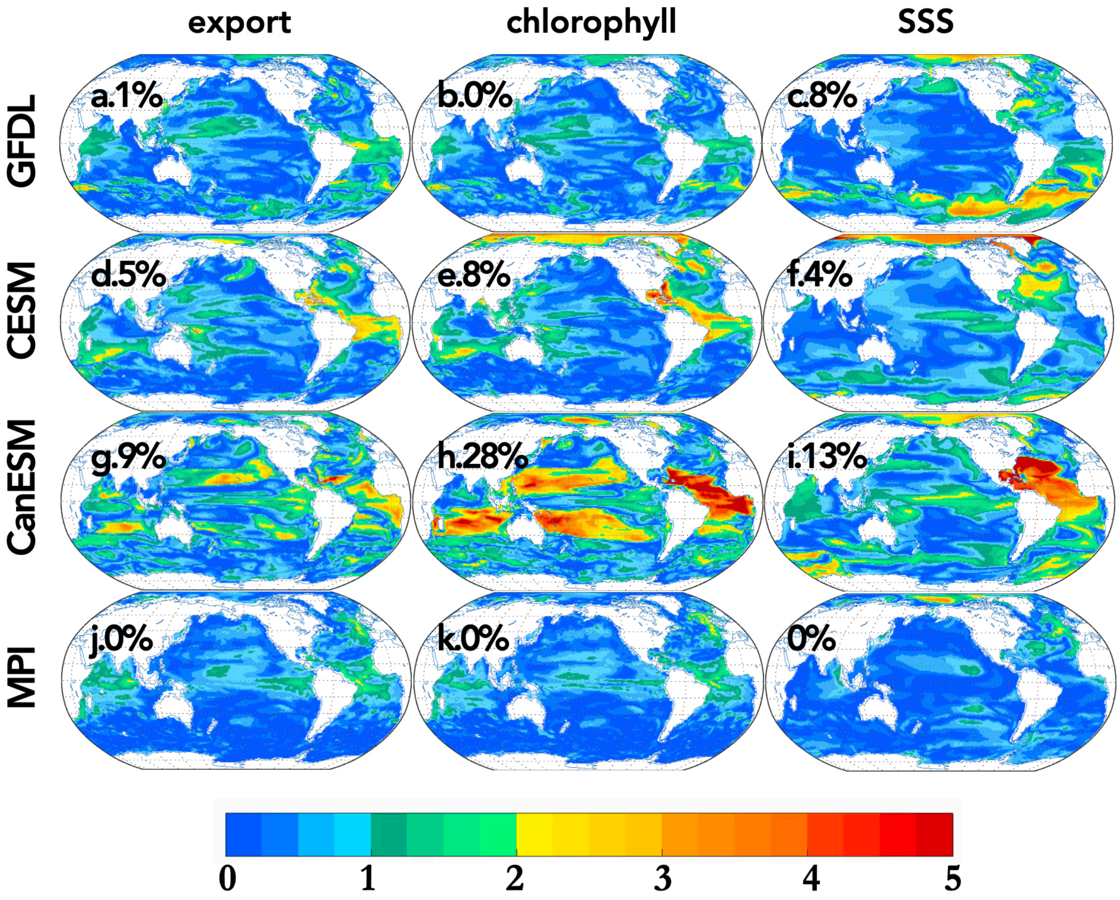

Figure S14. Maps of **Signal-to-Noise Ratio** for Export the four Large Ensembles for Export, Chlorophyll and SSS. For export production and chlorophyll the SNR is given for the 30-year period 1998-2027. For sea surface salinity the SNR is given for the 30-year period 2000-2029. The percent of ocean area with SNR > 2 shown on upper right corner of each map. The multi- LE mean Global SNR given for each variable at bottom of their respective columns.

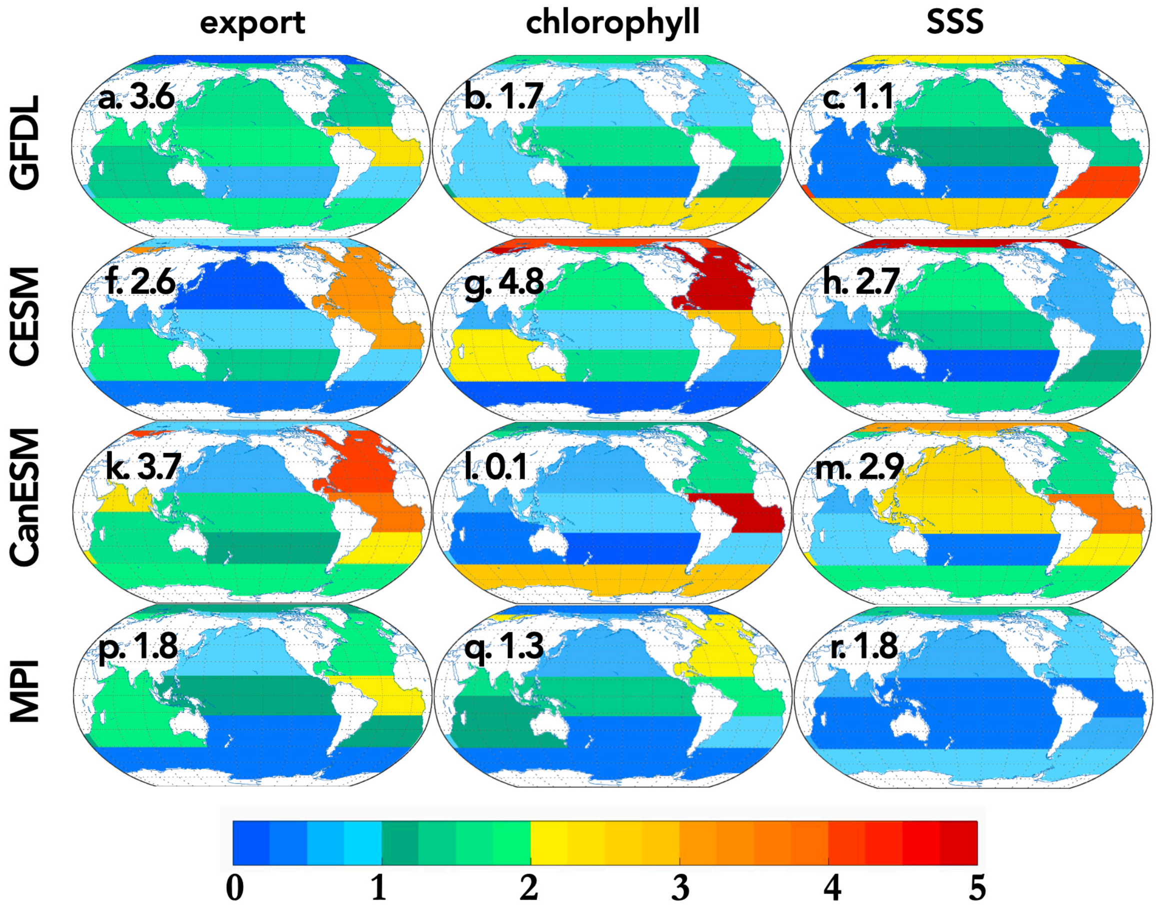

Figure S15. Maps of **regional Signal-to-Noise Ratio** for Export the four Large Ensembles for Export, Chlorophyll and SSS. For export production and chlorophyll the SNR is given for the 30-year period 1998-2027. For sea surface salinity the SNR is given for the 30-year period 2000-2029. Each LEs Global SNR ratio given on upper left corner of map for each variable.

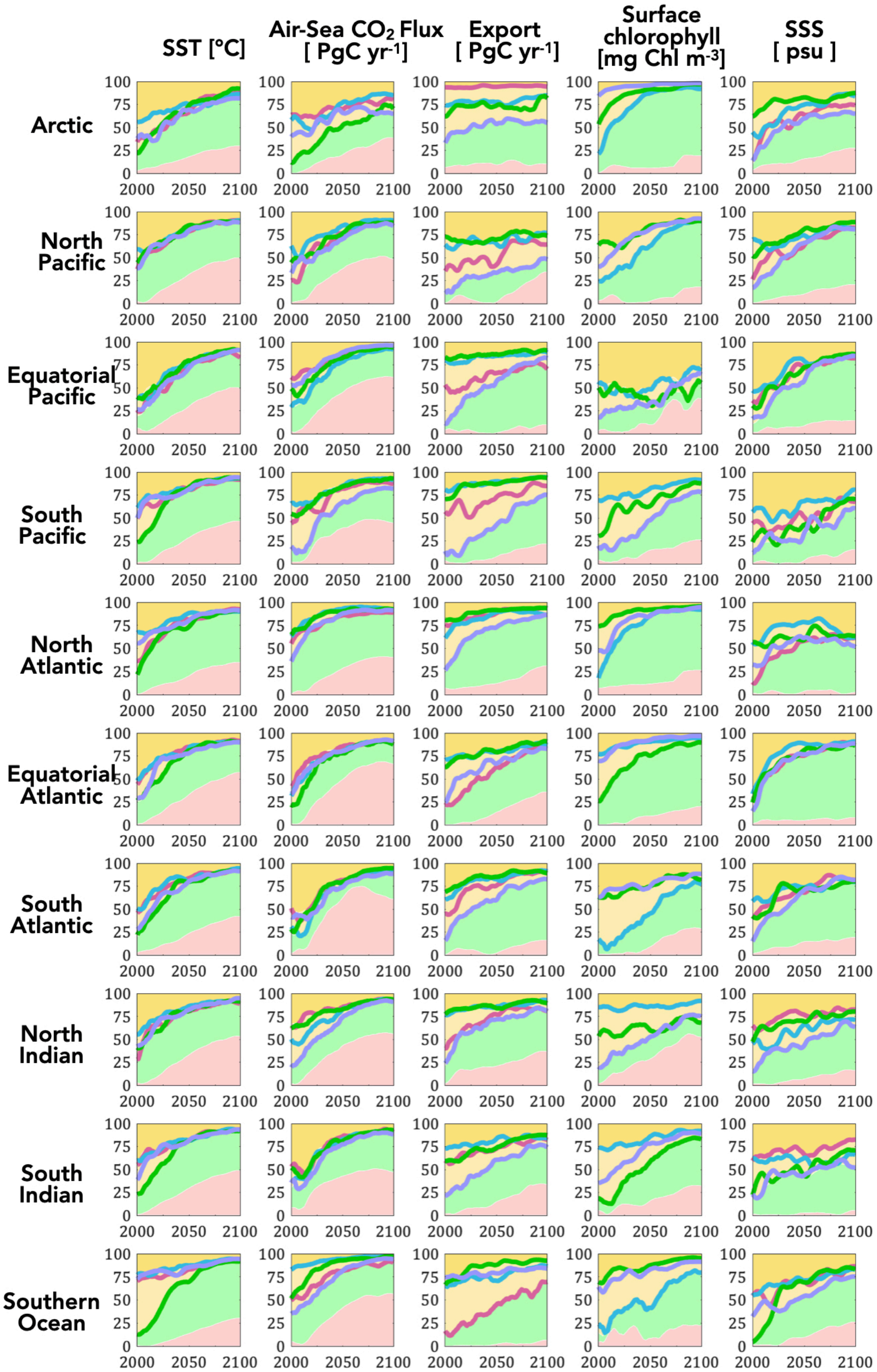

Figure S16. Partitioning uncertainty for a. SST, b. air-sea CO_2 Flux, c. Export, d. Chlorophyll and SSS, for scenario uncertainty (red, RCP4.5 vs RCP8.5), model uncertainty (green shading) and internal variability (yellow shading). The contribution of natural variability from each ensemble is given by the colored lines, same model-color relation as previous figures. The interface between yellow and green is determined by maximum contribution from internal variability, i.e. the model with the largest internal variability at that point in time. Ten year box filter was applied to the estimates of internal variability.
